# Uncertainty–Aware Prediction and Multi–Objective Optimization of Copper Smelting Performance Using Industrial Production Data

**DOI:** 10.3390/ma19214481

**Published:** 2026-10-24

**Authors:** Xiaoxue Zhang, Kai Yang, Jianxin Pan, Wuliang Yin, Qingtai Xiao, Gang Yu

**Affiliations:** 1 State Key Laboratory of Complex Nonferrous Metal Resources Clean Utilization, School of Metallurgical and Energy Engineering, Kunming University of Science and Technology, Kunming 650032, China; zhangxxwssy@163.com (X.Z.); kaiyang95@163.com (K.Y.); 2 State Key Laboratory of Intelligent Optimized Manufacturing in Mining & Metallurgy Processes, BGRIMM Technology Group, Beijing 102600, China; 3 Southwest United Graduate School, Kunming 650092, China; 4 Guangdong Provincial Key Laboratory of Interdisciplinary Research and Application for Data Science, Faculty of Science and Technology, Beijing Normal–Hong Kong Baptist University, Zhuhai 519088, China; jianxinpan@uic.edu.cn; 5 Department of Electrical and Electronic Engineering, School of Engineering, The University of Manchester, Manchester M13 9PL, UK; wuliang.yin@manchester.ac.uk

**Keywords:** copper smelting, data–driven modeling, uncertainty quantification, hybrid learning, multi–objective optimization

## Abstract

**Highlights:**

Intelligent prediction of copper smelting performance is achieved using industrial production data. A hybrid learning model captures nonlinear relationships among metallurgical variables. Uncertainty quantification improves the reliability of process optimization decisions. Multi–objective optimization balances copper enrichment, impurity control, and slag stability. The framework supports feed–composition assessment and adjustment in copper smelting.

**What are the main findings?**

- A hybrid learning framework accurately predicts key copper smelting performance indicators.
- Prediction uncertainty is quantified and incorporated into the optimization process.
- Multi–objective optimization identifies robust operating regions for metallurgical production.

**What are the implications of the main findings?**

- The framework supports data–driven feed–composition decisions in copper smelting.
- Reliable predictions improve the application of data–driven methods in industry.
- The strategy provides insights for balancing product quality and process stability.

**Abstract:**

Accurate prediction and reliable optimization of metallurgical processes are essential for improving the efficiency and stability of copper smelting under complex operating conditions. This work develops an uncertainty–aware hybrid learning framework to predict copper smelting indicators from feed composition and support multi–objective feed–composition optimization. The framework integrates a Transformer–based multi–task uncertainty model (TMUM) with target–specific histogram–based gradient boosting regressors (HGBR), where bias correction, variance calibration, and uncertainty propagation are introduced to obtain reliable, fused predictions. The calibrated surrogate model is further coupled with a reference–vector–guided evolutionary algorithm (RVEA) to optimize multiple process objectives while considering prediction uncertainty. The proposed TMUM–HGBR model achieves coefficients of determination of 0.8317, 0.8824, and 0.8373 for three key indicators, with 95% prediction interval coverage probabilities of 93.6%, 92.7%, and 94.1% on the independent test set. Random 5–fold cross–validation confirmed the stability of the model, and a prospective evaluation on newly collected production data, with the prediction state updated from available laboratory results, showed that the model tracked all three indicators. The optimization results reveal a trade–off between copper enrichment and impurity reduction, with predicted slag basicity remaining close to its historical reference. The framework provides data–driven decision support for feed–composition adjustment within the studied production conditions.

## 1. Introduction

Copper is an important base metal widely used in power, electronics, and advanced manufacturing. Declining ore grades, fluctuating feed compositions, and stricter environmental requirements have imposed increasingly complex control demands on copper smelting. Accurate prediction and collaborative optimization of key process indicators under variable raw–material and operating conditions are therefore essential for intelligent and efficient production. In practice, copper smelting requires simultaneous consideration of metal recovery, environmental constraints, and process stability [1,2,3]. Traditional control relies mainly on empirical rules, mechanistic analysis, and manual parameter tuning [4,5,6], which often struggle with high–dimensional, nonlinear, and noisy industrial data [7,8,9], particularly when multiple coupled objectives must be considered [10]. Mechanistic models are also limited by process complexity and parameter identifiability, restricting real–time applicability [10,11,12]. Copper recovery, heavy–metal migration, and slag basicity jointly determine the economic, environmental, and operational performance of the smelting process [13,14], making key–indicator modeling and control a typical multi–objective optimization problem.

Data–driven approaches provide an alternative by extracting useful information from historical plant data for prediction and operational decision–making. Data–driven learning has also shown potential for process regulation under nonlinear and nonrepetitive uncertainties [15]. Recent studies have further demonstrated that uncertainty–aware machine learning models can provide both accurate predictions and quantitative reliability estimates for complex manufacturing processes [16]. Legacy metallurgical data have been successfully reused for plant–performance analysis and process optimization [17], while causal discovery and probabilistic forecasting methods have improved nonlinear time–series modeling and uncertainty characterization [18,19]. For multi–output soft sensing, Wan et al. (2024) developed an attention–augmented broad random forest [20]. The causal and topology–informed graph neural networks have been introduced to improve robustness, causal consistency, and data efficiency [21,22]. Explainable soft sensing has also been investigated through causal feature selection and SHAP analysis [23].

Data–driven modeling has increasingly been applied in mineral processing to capture nonlinear relationships among material properties, operating variables, and process performance [24]. In industrial soft sensing, Wang et al. (2024) developed a deep–learning method that preserves local spatiotemporal structure for quality–variable prediction in complex industrial processes [25]. Related advances include Temporal Fusion Transformers for interpretable forecasting [26], reinforcement learning incorporating process constraints and control objectives [27], target–related Transformer networks for industrial quality prediction [28], and knowledge–refined hybrid graph models integrating spatiotemporal information and domain knowledge [29]. Hybrid and interpretable strategies have also demonstrated advantages in industrial thermal–process prediction [30], while mechanistic–LSTM models and adaptive distribution–learning networks have improved prediction stability and generalization under complex dynamics and covariate shifts [31,32]. Hybrid modeling strategies that combine different modeling paradigms have also been reported to improve prediction robustness by integrating complementary information sources [33]. In materials informatics, Hussain et al. (2026) introduced MultiMMNet for composition–based property prediction with uncertainty estimation [34], while Ullah et al. (2026) combined differential attention with graph neural networks for crystal–structure–based property prediction [35]. These studies highlight the potential of attention mechanisms and model integration for learning composition–property relationships. However, their applicability to process–dependent smelting indicators requires validation using industrial production data.

Data–driven modeling has also progressed in copper smelting and related nonferrous metallurgy. He et al. (2023) developed a dynamic soft sensor for online matte–grade prediction in oxygen–enriched side–blown bath smelting [36]. Tan et al. (2025) combined data decomposition with machine learning for cobalt–ion prediction in zinc hydrometallurgy [37]. Ouyang et al. (2026) developed a Transformer–based multi–task framework for simultaneous endpoint and composition prediction in copper converting [38], and Zou et al. (2025) further proposed a knowledge–guided encoder–decoder network for online matte–grade estimation in flash smelting [39]. Zhang et al. (2026) developed a Bayesian–optimized XGBoost framework for joint prediction of coal and silica addition requirements in copper bath smelting [40].

Despite progress in hybrid modeling and multi–task learning [41,42,43], their application to copper–smelting decision support requires careful consideration of target–dependent predictive behavior, relationships among process indicators, and competing optimization objectives. Industrial data are also affected by noise, outliers, missing values, skewed distributions, limited sample sizes, operating–condition changes, and distribution shifts, which can impair model robustness and generalization [44,45]. Recent CNN–Transformer stacking ensembles have also been explored to improve temporal robustness and generalization in metallurgical prediction [46]. Distribution–aware soft sensing has attempted to alleviate performance degradation under time–varying operating conditions through temporal distribution analysis and representation alignment [47], but reliable cross–condition prediction remains challenging. In addition, different targets may exhibit distinct statistical characteristics and formation mechanisms, making it difficult for a single model to perform consistently across all outputs [48,49,50]. More importantly, insufficient uncertainty quantification can make subsequent optimization highly sensitive to prediction errors and reduce the reliability of recommended operating schemes [51,52,53]. Uncertainty–aware optimization frameworks have recently been investigated in materials and manufacturing applications to avoid unreliable solutions caused by prediction errors and limited data availability [54]. Although machine–learning–assisted optimization has shown promise in mineral processing, uncertainty–aware optimization frameworks that explicitly account for prediction reliability remain limited.

Motivated by the broader concerns of resource utilization, environmental protection, and slag–system stability in copper smelting, this work focuses on three composition–based indicators: the copper grade enrichment ratio, the composite matte impurity index, and slag basicity. Studies on copper–slag treatment have investigated the recovery of copper and other valuable elements through physical separation, pyrometallurgical treatment, and hydrometallurgical routes [55,56,57]. Research on molten slags has demonstrated that basicity influences density, surface tension, and slag structure [58], while studies in steel refining have examined the effects of slag basicity and composition on inclusion evolution, desulfurization, and steel cleanliness [59,60]. These findings provide broader metallurgical context for considering metal enrichment, impurity levels, and slag chemistry together, although their quantitative relationships depend on the specific process and slag system. The three indicators employed in this work mainly describe the specific component characteristics in the smelting performance. Their joint prediction and use in optimization motivate the development of a modeling framework that combines nonlinear prediction, model complementarity, and explicit consideration of prediction uncertainty.

Accordingly, this work proposes a fusion framework integrating a Transformer–based Multi–task Uncertainty Model (TMUM) and a Histogram–based Gradient Boosting Regressor (HGBR) for multi–objective prediction in copper smelting. Bias and variance calibration are incorporated to quantify prediction credibility, and the resulting uncertainty–aware surrogate model is combined with a reference–vector–guided evolutionary algorithm (RVEA) for robust multi–objective optimization. Interpretability analysis is further employed to examine feature contributions and model behavior, while an interactive system integrates data preprocessing, prediction, uncertainty evaluation, and process optimization. The proposed TMUM–HGBR–RVEA framework therefore provides an integrated route from multi–indicator prediction to uncertainty–aware operational decision–making in copper smelting.

The remainder of this work is organized as follows. Section 2 presents the methodology, including data preprocessing, TMUM–HGBR modeling, uncertainty calibration, interpretability analysis, and RVEA–based multi–objective optimization. Section 3 describes the industrial dataset and preprocessing procedures. Section 4 presents the prediction, uncertainty, interpretability, optimization, and system–validation results. Section 5 concludes the work.

## 2. Methods

### 2.1. Model Fusion and Uncertainty Calibration

To achieve accurate prediction of multi–objective metallurgical process indicators while accounting for non–negativity and prediction uncertainty, a fusion model is constructed based on two base learners, TMUM and HGBR. Bias and variance calibration and uncertainty–aware fusion were completed on the validation set. Non–negativity post–processing was applied on the original physical scale, and interval estimates were provided. Transformer encoder was used as the core component. A non–negativity soft penalty and heteroscedastic uncertainty estimation were introduced to build an end–to–end trainable TMUM. The model serialized input features into embeddings, from which the Transformer encoder extracted global features. A dual–branch output head then generated the predicted mean and the log variance simultaneously. The model was optimized end–to–end using a weighted negative log–likelihood (NLL) loss as the main objective, combined with a non–negativity soft penalty and a variance regularization term. The input feature vector $x\in R^{d_{\mathrm{input}}}$ was mapped per feature through a trainable embedding matrix to obtain an embedding sequence. Each feature was treated as an independent token, allowing the Transformer to capture interactions between features. The sequence was fed into an L–layer Transformer encoder with multi–head self–attention, a feed–forward network, residual connections, layer normalization, and dropout. The sequence from the final encoder layer was average–pooled along the sequence dimension to obtain a global feature vector. After dropout, two parallel fully connected branches mapped the global feature. The mean branch outputs *μ* ∈ ℝ*^N^* and the log–variance branch outputs log*σ*^2^ ∈ ℝ*^N^*. The standard deviation $\sigma =\exp\left(\frac{1}{2}\log\sigma^{2}\right)$ ensures non–negative variance.

The heteroscedastic Gaussian likelihood as the modeling assumption, considering that the predicted target variable for each sample approximately follows a conditional Gaussian distribution given the input. The NLL loss was defined in

(1) $$L_{\mathrm{nll}}=\frac{1}{2}\left(\frac{{\left(y-\mu \right)}^{2}}{\sigma^{2}}+\log\sigma^{2}\right)$$

where *y* refers to the true target value of the sample. The NLL loss served as the main supervisory signal for the model. To discourage negative predictions, an L2–based non–negativity soft penalty was applied in the normalized output space. It can be expressed as

(2) $$L_{\mathrm{phys}}=E\left[\sum_{j=1}^{N}\mathrm{ReLU}{\left(-\mu_{j}\right)}^{2}\right]$$

where *E*[·] represents the expected value of the current batch of samples, *N* is the total number of the predicted targets. This penalty did not rely on the training set boundaries and was differentiable and easy to optimize. To prevent numerical instability caused by excessive growth of the log variance, an L1 regularization term on the log variance was introduced

*L*_unc_ = 𝔼[∣log*σ*^2^∣] (3)

The total loss was expressed as

*L* = *L*_nll_ + *λ_p_L*_phys_ + *λ_u_L*_unc_ (4)

where the hyperparameters were fixed as *λ_p_* = 0.03 and *λ_u_* = 0.02. The model used the AdamW optimizer. The initial learning rate and weight decay were determined through optuna hyperparameter search. During training, the total loss on the validation set was monitored. Training was terminated if no improvement was observed for a patience of 20 consecutive epochs, and the best model parameters were restored.

HGBR is a highly efficient regression algorithm based on gradient boosting decision trees. Because multi–target features differ in noise level, nonlinearity, and interaction structure, using a single model to fit multiple targets simultaneously inevitably introduces a statistical compromise among targets and impairs generalization performance. Therefore, separate regressors were trained for each target, allowing the model complexity and regularization strength to adaptively match the characteristics of each target. Each single–target model used squared error loss and standard gradient boosting. To suppress overfitting, the model introduced leaf–level L2 regularization and a minimum leaf sample constraint. For HGBR, hyperparameter optimization was performed independently for each target using three–fold cross–validation within the training set, and each model was subsequently refit on the full 702–sample training set using the selected optimal hyperparameters.

**Figure 1:**
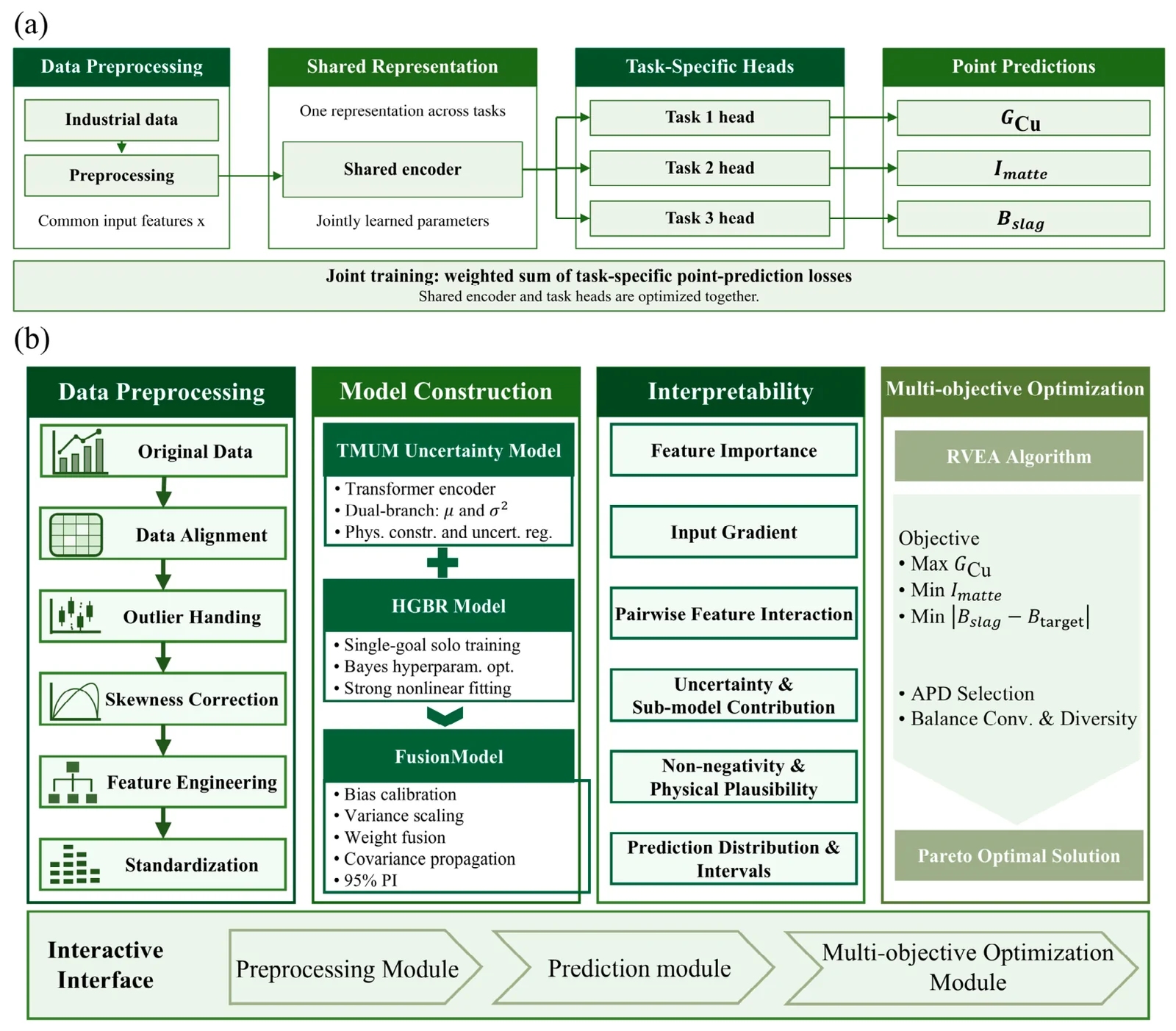
Overall framework. (**a**) Conventional multitask learning; (**b**) the proposed TMUM–HGBR–RVEA framework.

Conventional MTL generally shares a single encoder across all tasks and minimizes a weighted sum of point–prediction losses, so that all targets are forced to share one representation, and only deterministic outputs are produced. The proposed framework differs from this paradigm in four respects. First, the shared Transformer encoder is used only inside TMUM to capture cross–feature interactions, while the dual–branch output head produces a task–specific mean and log variance for each target; the model is trained with a heteroscedastic Gaussian NLL loss together with a non–negativity penalty and a variance regularization term, so that the noise level and the physical plausibility of each target are modeled explicitly. Second, the targets are not forced through a single network: an independent HGBR is trained for each target to capture target–specific nonlinearity and interaction structure, and the two heterogeneous learners are combined rather than sharing parameters. Third, the combination is performed by a calibrated fusion on the original physical scale, in which the biases and the variance scaling factor are estimated from validation residuals, a constant fusion weight is solved by minimizing the squared error, and the residual covariance between the two learners is propagated, yielding a calibrated predictive distribution rather than a point estimate. Fourth, the calibrated distribution is carried into the subsequent decision–making, where the uncertainty penalty in RVEA favors robust feed compositions and the interpretability analysis is performed on the fused model. Figure 1a,b contrast the two paradigms: in conventional MTL, the shared encoder and the task–specific heads are optimized jointly for point prediction, whereas in the proposed framework the shared trunk is confined to TMUM and the target–specific HGBR, the calibration layer and the uncertainty–aware optimization lie outside the shared representation.

For evaluating the predictive performance and uncertainty quantification in copper smelting process prediction, five main metrics were employed: the coefficient of determination (*R*^2^), root mean square error (RMSE), mean absolute error (MAE), 95% prediction interval coverage probability (PICP_95_) and mean 95% prediction interval half–width (MPIHW_95_). *R*^2^ measures the goodness of fit, with values closer to one indicating stronger agreement between the predicted and observed values. RMSE quantifies the overall magnitude of prediction errors, whereas MAE represents the mean absolute deviation between predictions and observations; lower RMSE and MAE indicate better predictive accuracy. For the i–th sample, the calibrated 95% prediction interval is defined as $\left[L_{i},U_{i}\right]=\left[{\hat{y}}_{i},-1.96{\hat{\sigma }}_{i}{\hat{y}}_{i}+1.96{\hat{\sigma }}_{i}\right]$, where ${\hat{y}}_{i}$ and ${\hat{\sigma }}_{i}$ represent the predicted mean and predicted standard deviation, respectively. PICP_95_ represents the proportion of true observations that fall within the 95% prediction interval, and can be expressed as

(5) $${\mathrm{PICP}}_{95}=\frac{1}{N}\sum_{i=1}^{N}I\left(L_{i}\leq y_{i}\leq U_{i}\right)\times 100\%$$

The closer its value is to the nominal coverage level of 95%, the better the uncertainty calibration effect of the model is. MPIHW_95_ is used to measure the compactness of the prediction interval, and it is calculated as

(6) $${\mathrm{MPIHW}}_{95}=\frac{1}{N}\sum_{i=1}^{N}\frac{U_{i}-L_{i}}{2}$$

When the coverage rate is similar, the smaller the MPIHW_95_ is, the more concentrated the prediction interval will be. To evaluate the calibration over multiple nominal levels, the empirical coverage was computed at nominal levels from 50% to 99%, and the mean absolute calibration error (ACE) and the root–mean–square calibration error (RMSCE) were defined as the mean absolute deviation and the root–mean–square deviation between the empirical coverage and the nominal level over these levels, respectively. Split conformal calibration with normalized nonconformity scores was further applied as a benchmark: the conformal quantile ${\hat{q}}^{\alpha }$ was estimated on the validation set as the [(*n* + 1)*α*]/*n* empirical quantile of the normalized absolute residuals $\left|y_{i}-{\hat{y}}_{i}\right|/\sigma_{i}$, and the corresponding interval was constructed as ${\hat{y}}_{i}\pm {\hat{q}}_{\alpha }\sigma_{i}$.

Fully utilizing the complementary advantages of TMUM and HGBR, a fusion strategy was implemented on the original physical scale. Bias correction, uncertainty calibration, and fusion weight estimation were all performed using the validation set. The expected biases of TMUM and HGBR were estimated from their validation residuals and were subsequently removed from the corresponding predictions. A variance scaling factor for TMUM was estimated from its validation residuals to calibrate the predicted heteroscedastic variance. The scaling factor was computed as the ratio of the mean squared error of the TMUM on the validation set to the expected value of its output variance. The calibrated TMUM prediction variance was the original variance multiplied by this factor. Based on the validation set data, a constant weight that minimized the sum of squared errors between the weighted prediction and the true value was solved, and the weight was clipped to the interval [0, 1]. The final fusion prediction for target *Y* of sample *i* as

(7) $${\hat{y}}_{\mathrm{iY}}^{\mathrm{fus}}=\alpha_{Y}^{*}{\hat{y}}_{\mathrm{iY}}^{\mathrm{TX}}+\left(1-\alpha_{Y}^{*}\right){\hat{y}}_{\mathrm{iY}}^{\mathrm{SP}}$$

where $\alpha_{Y}^{*}$ is the optimal fusion weight solved independently for target *Y*, ${\hat{y}}_{\mathrm{iY}}^{\mathrm{TX}}$ refers to the predicted value of the target Y for sample i in TMUM. ${\hat{y}}_{\mathrm{iY}}^{\mathrm{SP}}$ refers to the predicted value of the target *Y* for sample *i* in the HGBR model. The HGBR residual variance and the residual covariance between TMUM and HGBR were estimated from the validation residuals and propagated into the fused prediction. The resulting fusion variance for each sample was calculated from the calibrated TMUM variance, the HGBR residual variance, and the covariance between the two base models according to the fusion weights. After fusion, negative predictions were clipped to zero as a simple non–negativity post–processing step.

### 2.2. Hyperparameter Optimization and Interpretability

Hyperparameter optimization was a key step in determining the final performance of the model. To achieve an optimal trade–off among model capacity, regularization strength, non–negativity regularization, and uncertainty modeling, the tree–structured Parzen Estimator (TPE) sampler of optuna was employed to conduct Bayesian optimization on the key hyperparameters of the two base learners, TMUM and HGBR. For TMUM, the objective function of hyperparameter optimization was the total loss on the validation set. This loss consisted of a weighted sum of three parts. The first part was the weighted Gaussian NLL of the targets in the output scaling space. The second part was the non–negativity soft penalty. The third part was the uncertainty regularization term. In each trial, the training phase used the same total loss for gradient updates, enabling the model to learn representations while discouraging negative predictions and adapting to heteroscedastic noise. Optimization was performed for 60 iterations with the validation loss as the objective. In each search round, the model was trained for a maximum of 200 epochs. Early stopping based on the validation loss was applied with a patience of 20 epochs. The model state with the lowest validation loss was selected. For HGBR, hyperparameter optimization was performed independently for each output target *Y*. The optimization objective was the mean RMSE from threefold cross–validation. The configuration with the smallest cross–validation RMSE was selected as the optimal hyperparameter for that target. Twenty trials were conducted for each target. The validation set was kept separate from HGBR model fitting and was used for Transformer hyperparameter selection and early stopping, bias and uncertainty calibration, and fusion model selection and calibration, while the independent test set was used only for final performance and uncertainty evaluation.

To gain a deep understanding of the prediction behavior of the fusion model, verify its rationality, and enhance trust in the decision–making process, an interpretability analysis was conducted. The analysis covered feature importance, input gradients, pairwise feature interactions, uncertainty decomposition, and non–negativity and prediction plausibility assessment. Permutation feature importance was used to evaluate the global impact of each feature on the fusion prediction. For each feature, its values in the test set were randomly shuffled multiple times. The degradation in fusion prediction performance relative to the original prediction was then calculated after each shuffle. The importance of feature j was defined as the average performance degradation over *R* repetitions.

(8) $$I_{j}=\frac{1}{R}\sum_{r=1}^{R}\left[M\left(y_{\mathrm{true}},{\hat{y}}_{\mathrm{fused}}^{\left(r\right)}\right)-M\left(y_{\mathrm{true}},{\hat{y}}_{\mathrm{fused}}^{\mathrm{base}}\right)\right]$$

where ${\hat{y}}_{\mathrm{fused}}^{\mathrm{base}}$ is the fusion prediction on the original test set and ${\hat{y}}_{\mathrm{fused}}^{\left(\mathrm{perm}\right)\left(r\right)}$ is the prediction after permuting feature *j* in the *r* repetition. This analysis produced an importance matrix of dimension [*n*_features_, *n*_targets_], supporting target–specific feature ranking. Input gradient analysis examined the sensitivity of the fusion output to input features from a local perspective. Because the fusion was a linear combination of the TMUM outputs, the gradient of the fusion prediction with respect to the original input *x*_orig_ for target *k* was calculated by

(9) $$\frac{\partial {\hat{y}}_{\mathrm{fused},k}}{\partial x_{\mathrm{orig}}}=\alpha_{k}\cdot \frac{\partial {\hat{y}}_{\mathrm{tx},k}}{\partial x_{\mathrm{orig}}}$$

Because the input–gradient analysis describes the local sensitivity of the fusion output, it does not by itself establish the presence of feature interactions. Pairwise interactions were therefore quantified with Friedman’s H–statistic, which measures the deviation of the joint partial dependence of two features from additivity

(10) $$H_{\mathrm{jk}}^{2}=\frac{\sum_{i=1}^{N}{\left[{\mathrm{PD}}_{\mathrm{jk}}\left(x_{\mathrm{ij}},x_{\mathrm{ik}}\right)-{\mathrm{PD}}_{j}\left(x_{\mathrm{ij}}\right)-{\mathrm{PD}}_{k}\left(x_{\mathrm{ik}}\right)\right]}^{2}}{\sum_{i=1}^{N}{\mathrm{PD}}_{\mathrm{jk}}{\left(x_{\mathrm{ij}},x_{\mathrm{ik}}\right)}^{2}}$$

where PD*_jk_* is the two–dimensional partial dependence of the target on features j and *k*, PD*_j_* and PD*_k_* are the corresponding one–dimensional partial dependences, and i indexes the evaluation samples. For each pair, the two features were varied over a five–point grid spanning the 10th–90th percentiles of their training–set values, while the remaining features were held at their observed values for *N* = 200 samples randomly selected from the test set. *H_jk_* ranges from 0 to 1, and a larger value indicates a stronger non–additive interaction.

The procedure was applied to all 91 feature pairs for each of the three targets. To verify the stability of the ranking, the computation was repeated with grid sizes of four to six points, sample sizes of 150 and 200, and different random seeds; the five strongest pairs of each target remained unchanged. The H–statistic is therefore used as the interaction measure in the following analysis, whereas the gradient–based quantities are interpreted only as local sensitivities.

Uncertainty decomposition quantified the variance contributions from different sources in the fusion prediction. The TMUM output variance $\sigma_{\mathrm{tx},\mathrm{orig}}^{2}$ was obtained by scaling its log variance through the target normalizer. The HGBR variance $\sigma_{\mathrm{sp},\mathrm{orig}}^{2}$ was estimated from the training residual variance. The covariance of the two models, Cov_resid_, was also estimated using the validation residuals. The total variance of the fusion prediction was calculated by

(11) $$\sigma_{\mathrm{fused}}^{2}=\alpha^{2}\sigma_{\mathrm{tx},\mathrm{orig}}^{2}+{\left(1-\alpha \right)}^{2}\sigma_{\mathrm{sp},\mathrm{orig}}^{2}+2\alpha \left(1-\alpha \right){\mathrm{Cov}}_{\mathrm{resid}}$$

where *α* is a vector of fusion weights and the multiplications are performed element–wise. Furthermore, the percentage contributions of the Transformer, HGBR, and the covariance term to the total variance were calculated, thereby clarifying which component dominated the uncertainty. Non–negativity and prediction plausibility assessment were used to examine whether the predicted values satisfied the basic non–negative requirement and exhibited reasonable statistical characteristics. The number and proportion of samples that violated the constraints on the test set were counted, and basic statistics of the predicted values were reported to assess whether the model outputs remained within plausible ranges.

### 2.3. Multi–Objective Optimization with RVEA

A multi–objective feed–composition optimization framework was constructed using the trained fusion surrogate model. The parameter configuration of the RVEA was as follows. The population size was set to 50 with 91 reference directions, and the maximum number of generations was 30. Simulated binary crossover was used for crossover, with a crossover probability of 0.9 and a distribution index of 15. Polynomial mutation was used for mutation, with a distribution index of 20. The selection process employed an elite preservation strategy based on the APD, retaining the individual with the smallest APD in the direction of each reference vector. The initial population was randomly generated within the decision variable bounds using FloatRandomSampling. To ensure reproducibility of the experiments, a fixed random seed was set for all stochastic processes. These parameter settings were determined through preliminary sensitivity analysis, balancing computational efficiency with optimization quality.

The decision vector *x* = [*x*_1_, *x*_2_, …, *x_n_*] comprises the feed–composition variables, with *x_i_* denoting the concentration of the corresponding feed component. The objective functions were defined as

min{−, *f*_1_, (*x*)*f*_2_(*x*)∣*f*_3_(*x*) − *B*_target_∣} (12)

where *f*_1_(*x*), *f*_2_(*x*), and *f*_3_(*x*) are the predicted values, respectively, obtained by feeding the input *x* into the trained fusion surrogate model. *B*_target_ represents the target alkalinity and is determined by calculating the mean value of the training set.

Constraint conditions were defined as

(13) $$x_{i}^{\min}\leq x_{i}\leq x_{i}^{\max},\;i=1,2,\ldots ,n$$

where $x_{i}^{\min}$ and $x_{i}^{\max}$ are the minimum and maximum values of the *i* feature in the historical data, respectively. These bounds ensure that the optimized solutions lie within feasible process operating ranges. In addition to the per–variable bounds, two joint constraints were imposed to restrict the search space to the historically feasible region. The total feed composition was constrained to the 1st–99th percentile range of the historical data (95.5–102.8 wt%). A robust distance from the training median, normalized by the interquartile range of each variable, was constrained to the 99.5th percentile of the historical distances (1.309). An optional composition–closure constraint further restricted the total composition to 100 ± 2 wt%. The RVEA outputs all nondominated solutions that constitute the Pareto front. The RVEA was used to search the solution space, and the historical solutions from each generation were collected. Different nondomination levels were obtained through fast nondominated sorting, where the first level corresponded to the Pareto front. After obtaining the Pareto front, a weighted scoring method was adopted to determine the Pareto compromise solution as the final recommended solution. The objective values were normalized to the interval [0, 1]. After the above processing, the three normalized indicators were all converted into positive evaluation indicators, meaning that a larger value indicates better comprehensive performance of the solution. If the range is zero, the normalized value is set to 0.5. The comprehensive score was calculated by

score = *ω*_1_ ⋅ *Y*_1,norm_ + *ω*_2_ ⋅ *Y*_2,norm_ + *ω*_3_ ⋅ *Y*_3,norm_ (14)

where *w*_1_, *w*_2_, and *w*_3_ respectively represent the scoring weights of the three objectives, and the sum of the weights is one. *Y*_(1, norm)_ refers to the normalized value of the target variable. The solution with the highest score was selected as the feed–composition.

Further enhancing the robustness of the optimization results, a prediction uncertainty penalty term was introduced into the objective function. For regions with high prediction uncertainty from the model, a penalty was applied during optimization, guiding the optimization algorithm to favor robust regions where the model was more confident. For each predicted point *μ*(*x*) from the prediction model, the corresponding prediction variance *σ*^2^(*x*) was also available. The uncertainty information was incorporated into the optimization objective function, which can be expressed as

(15) $${\tilde{F}}_{i}\left(x\right)=F_{i}\left(x\right)+\gamma \cdot \sigma_{i}\left(x\right),\;i=1,2,3$$

where ${\tilde{F}}_{i}\left(x\right)$ refers to the *i* objective value after considering uncertainty, *F_i_*(*x*) refers to the original predicted objective value, *σ_i_*(*x*) is the standard deviation of the prediction for the *i* objective from the prediction model, representing the prediction uncertainty at that point, and *γ* = 1 is the uncertainty weight coefficient that controls the strength of the uncertainty penalty, providing a moderate penalty that discourages high uncertainty regions without overly compromising original objective pursuit. By minimizing ${\tilde{F}}_{i}\left(x\right)$, the optimization process actively avoided decision points that had high model uncertainty even though their original performance predictions were good, while still pursuing the original performance objectives. The resulting Pareto front and the recommended solution had higher reliability, reducing the risk of poor actual performance caused by model prediction errors.

Figure 1 summarizes the overall architecture of the proposed method. Starting from raw industrial data, the framework performs outlier truncation, Yeo–Johnson skewness correction, feature engineering, and robust standardization. Based on TMUM, a dual–branch structure outputs a heteroscedastic Gaussian distribution, and a non–negativity soft penalty term along with an uncertainty regularization term is introduced, together with an HGBR trained independently for each target. On the validation set, a constant–weight fusion strategy is adopted for bias and variance calibration, and the covariance is propagated, ultimately yielding fused predictions with prediction interval half–width. The method analyzes feature importance, input gradients, pairwise feature interactions quantified by Friedman’s H–statistic, uncertainty decomposition, non–negativity, and prediction plausibility. Using the RVEA, a well–distributed Pareto front is generated, from which a trade–off optimal solution is selected via a weighted scoring method. This integrated framework provides data–driven prediction, uncertainty–aware decision–making, and robust multi–objective optimization for the copper smelting process.

## 3. Data and Preprocessing

**Figure 2:**
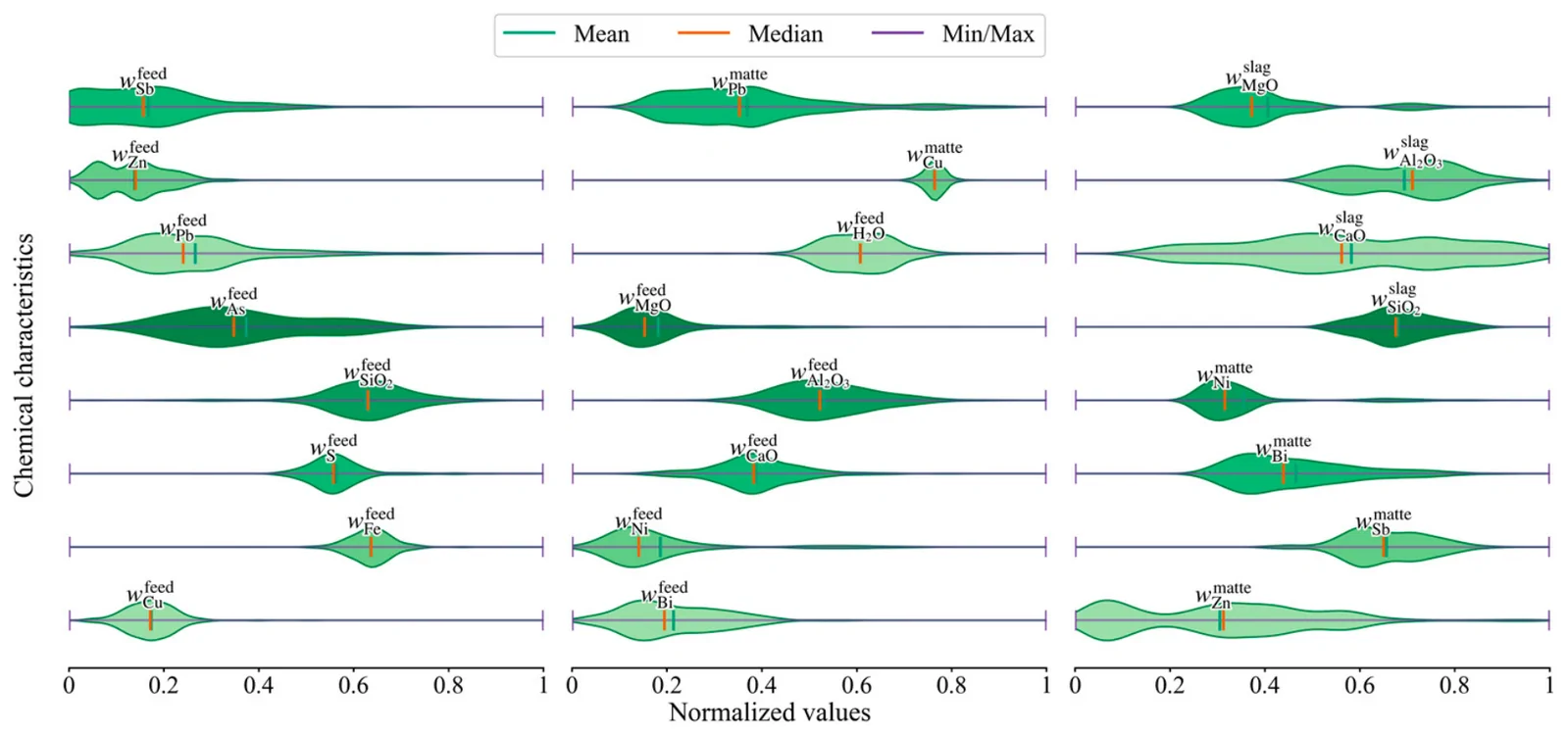
Original data structure.

The data used in this work are collected from the actual production process of a copper smelting plant. The raw material mixture is then sent to the smelting furnace. The burdening process is the focus and has a significant impact on the subsequent smelting process and results. The data consist of three types of records, namely mixed feed, copper matte, and slag. They respectively represent the raw material input, intermediate products, and slag output during the smelting process. After excluding the records lacking valid sampling time records, a total of 1149 batch mixed feed records, 3992 electric furnace copper matte records, and 1116 electric furnace slag records were obtained. The data collection intervals for the three types are not fixed. The median positive sampling intervals between adjacent valid records are 4.3 h, 1.2 h, and 5.8 h, respectively. There are a total of 24 variables, including 14 feed variables, 6 copper matte variables, and 4 slag variables. Table 1 provides detailed statistical characteristics of each variable, including the minimum, maximum, mean, and standard deviation. According to Figure 2, the distribution of each characteristic shows significant differences. Some are approximately symmetrical, while others exhibit right skewness, long tails, or multiple peaks. The composition of the raw materials and the production conditions are subject to fluctuations.

Because the sampling frequencies and sampling times of the mixed concentrate, copper matte, and slag records were inconsistent, product events were first constructed by slag–matte pairing. With each slag record as the reference, the matte record with the smallest time difference within a ±1.5 h window was selected, and the mean of their sampling times was taken as the event time, yielding 1116 product events. Subsequently, the feed–product lag τ was scanned from 0 to 24 h in 0.5 h steps. For each τ, feed records were matched by the nearest–neighbor principle within a fixed scanning window to events at t^feed^ + τ, subject to the causal constraint t^feed^ ≤ min(t^matte^,  t^slag^). The lag was determined by maximizing the composite score S(τ) = 0.45 R_chem_ + 0.45 R_pred_ + 0.10 R_cov_. S(τ) was obtained from the weighted percentile ranks of three metrics: the mean absolute Spearman correlation coefficient between feed and product chemical compositions, the feed–product composition prediction consistency, and the proportion of matched feed records. S(τ) reached its maximum of 0.911 at τ = 10 h and remained within 5% of the maximum over a 2.5 –h–wide interval containing 10 h, indicating that the optimum lies on a broad plateau rather than at an isolated point. At τ = 10 h, scanning matched 1107 feed records, with a coverage of 96.3%, a chemical consistency of 0.481, and a median time residual of 1.44 h. After shifting the product event times 10 h earlier, feed records were matched within a ±5 h window by the nearest–neighbor principle under the same causal constraint, finally yielding 1098 matched records with a coverage of 98.4%.

**Figure 3:**
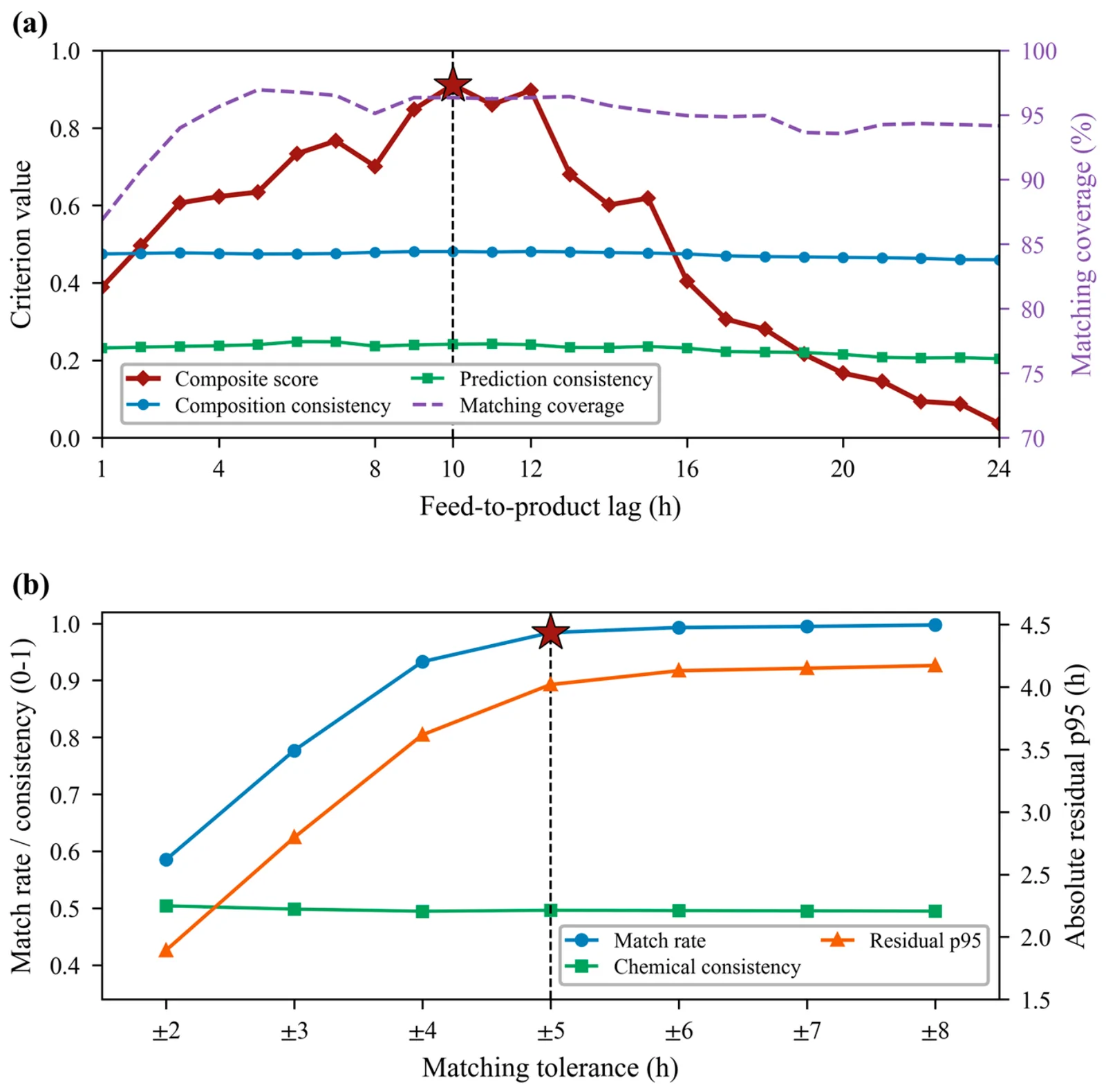
Sensitivity of time sequence matching. (**a**) Criterion values and coverage change with lag τ; (**b**) Matching rate, chemical consistency, and residual p95 change with tolerance Δ. The red stars indicate the selected optimal values.

It should be noted that the lag and tolerance were estimated from all records rather than from the training set alone. If estimated using only the training period, they would reflect only the sampling rhythm of that period, whereas the feed composition and sampling intervals in the validation and test periods differ. In addition, this criterion uses only the chemical consistency, prediction consistency, and matching coverage of paired records and does not involve the prediction errors of downstream models on the test set. To evaluate the impact of the above processing, the scan was rerun using only the training set, and the matched dataset was reconstructed using the resulting lag. The distribution of the matched target variable changed very little: the mean shift in *G*_Cu_ was <0.2%, and the maximum KS statistic was 0.026. Therefore, whether lag selection was performed within the training set had little effect on the matched data. Figure 3a presents the sensitivity of the matching results to the lag. Under a fixed scanning window, when the lag was varied from 4 to 12 h, the coverage remained at 95.1–96.8%, the chemical consistency at 0.475–0.481, and the median time residual at 1.2–1.5 h, and the number of matched records varied by no more than 2%. The ±5 h tolerance was slightly larger than the 95^th^ percentile of the residuals (4.0 h); further relaxing it to ±6 h increased the matching rate by only 0.9 percentage points but introduced marginal pairs with residuals of 5–6 h and did not improve the chemical consistency. Therefore, ±5 h was adopted. Figure 3b presents the tolerance sensitivity. With the lag fixed at 10 h and the tolerance varied from ±2 to ±8 h, the matching rate increased from 58.5% (±2 h) to 98.4% (±5 h) and then saturated (99.3% at ±6 h and 99.7% at ±8 h); the chemical consistency remained at 0.495–0.504, and the 95^th^ percentile of the absolute residuals increased from 1.9 to 4.2 h.

**Figure 4:**
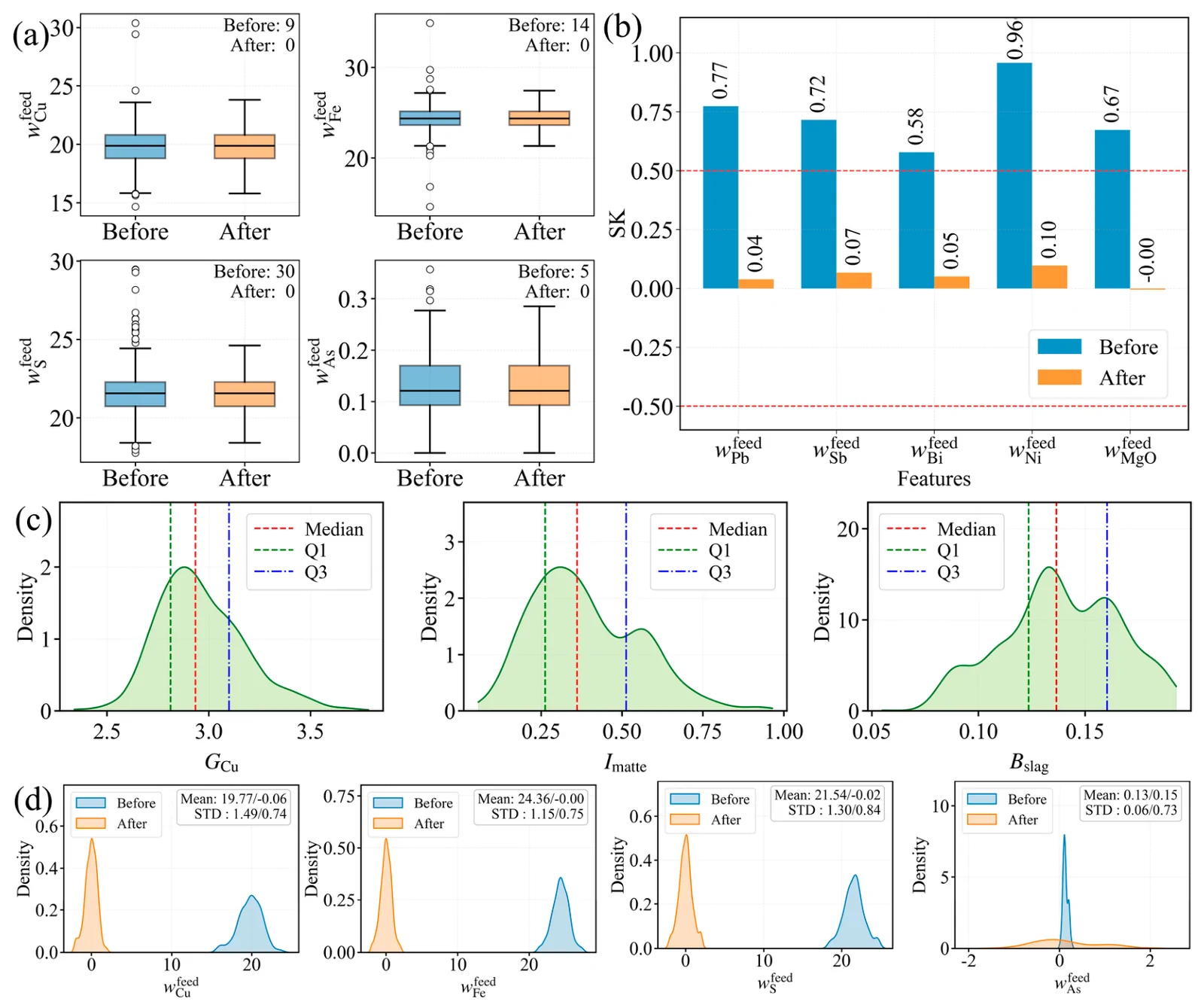
The pre-processing procedure. (**a**) Outlier truncation of representative feed–composition variables; (**b**) skewness of selected feed variables before and after Yeo–Johnson correction; (**c**) probability density distributions of the target variables; (**d**) distributions of representative input variables before and after robust standardization.

Strictly controlling information leakage, a preprocessing strategy of splitting first and then learning was adopted. First, the raw data were randomly split into a training set *X*_Train_ and a test set *X*_Test_ at a ratio of 8:2. Subsequently, all steps that required estimating parameters from data were fitted only on the training set, and the same parameters were then applied to the test set. The test set remained unseen during model development and was used only for final performance evaluation. To examine the stability of the model with respect to sample resampling, a random 5-fold cross-validation was additionally performed on the 878 samples of the training and validation portions, with the independent test set excluded. In each fold, four folds were used for training, one fold for testing, and 20% of the training portion for bias correction, fusion-weight estimation, and variance calibration. All stateful preprocessing steps were refitted within each fold, and the hyperparameters were fixed to the values obtained for the full model rather than re–tuned per fold. Figure 4 shows the effect of data preprocessing. To suppress the impact of extreme values on model training, a unified winsorization based on the interquartile range was applied to all numerical features. First, the first quartile *Q*_1*j*_ and the third quartile *Q*_3*j*_ for column *j* = 1, 2, …, 25 were calculated on the training set. The interquartile range was obtained as IQR*_j_* = *Q*_3*j*_ − *Q*_1*j*_. The lower and upper bounds were determined as *L_j_* = *Q*_1*j*_ − 1.5 × IQR*_j_* and *U_j_* = *Q*_3*j*_ + 1.5 × IQR*_j_*. Subsequently, the values in column *j* of the training, validation, and test samples, denoted as ${\hat{X}}_{\mathrm{Train}}$. The bounds were fitted only on the training set and then fixed for the remaining data splits. No samples were removed. The relative order of values within the bounds was preserved, and only the influence of outliers was reduced. Figure 4a shows that the trimming of the IQR removes the extreme values of the features $w_{\mathrm{Cu}}^{\mathrm{feed}},w_{\mathrm{Fe}}^{\mathrm{feed}},w_{S}^{\mathrm{feed}}$ and $w_{\mathrm{As}}^{\mathrm{feed}}$. Mitigating the impact of skewed distributions and heavy tails on the model, the sample skewness *SK* of each numerical feature was computed on the training set. Columns with ∣*SK*∣ > 0.5 were selected, and Yeo Johnson power transformation was fitted on the training subset $X_{\mathrm{ij}}^{\mathrm{Traincap}}$. Figure 4b shows that after processing the skewed data, all *SK* were reduced to below 0.5. Due to the high dimensionality of the output indicators, to simplify indicators and reduce difficulty of model construction and computational cost, multidimensional outputs were compressed into three derived indicators with clear metallurgical meanings are defined in

(16) $$\begin{array}{c} G_{\mathrm{Cu}}=\frac{w_{\mathrm{Cu}}^{\mathrm{matte}}}{w_{\mathrm{Cu}}^{\mathrm{feed}}}\\ I_{\mathrm{matte}}=\sum_{i=1}^{5}\omega_{i}\Phi_{i}\\ B_{\mathrm{slag}}=\frac{w_{\mathrm{CaO}}^{\mathrm{slag}}+w_{\mathrm{MgO}}^{\mathrm{slag}}}{w_{{\mathrm{SiO}}_{2}}^{\mathrm{slag}}+w_{{\mathrm{Al}}_{2}O_{3}}^{\mathrm{slag}}} \end{array}$$

where *G*_Cu_ is the matte-to-feed copper grade ratio, characterizing copper enrichment. *I*_matte_ is a composite index of the measured Zn, Ni, Pb, Bi, and Sb concentrations in matte, with Φ*_i_* denoting the robustly scaled concentration of element *i*. Entropy weighting summarizes their statistical variation across the training samples. Only on the training set, the 5th to 95th percentiles were calculated for the available harmful element $w_{\mathrm{Pb}}^{\mathrm{matte}},w_{\mathrm{Zn}}^{\mathrm{matte}},w_{\mathrm{Sb}}^{\mathrm{matte}},w_{\mathrm{Bi}}^{\mathrm{matte}},w_{\mathrm{Ni}}^{\mathrm{matte}}$, and robust scaling was applied to map them to [0, 1]. On the training set, a probability matrix was obtained by column wise normalization. The information entropy and deviation degree were then calculated and normalized into weights *ω_i_*. Table 2 shows their correspondences and data. The index is used to compare aggregate impurity levels within the studied dataset. *B*_slag_ reflects the basicity of the slag phase. Figure 4c shows the data structure of the output variables after feature engineering. Reducing the influence of outliers on scale estimation, a robust scaler was fitted separately on the training subset for the input features and the target features. The obtained parameters were then applied unchanged to the validation and test sets, thereby improving robustness to outliers and heavy tailed distributions while ensuring dimensional comparability. Figure 4d shows the changes in some features before and after standardization, robust scaling effectively reduces the dimensional and scale differences among features.

**Figure 5:**
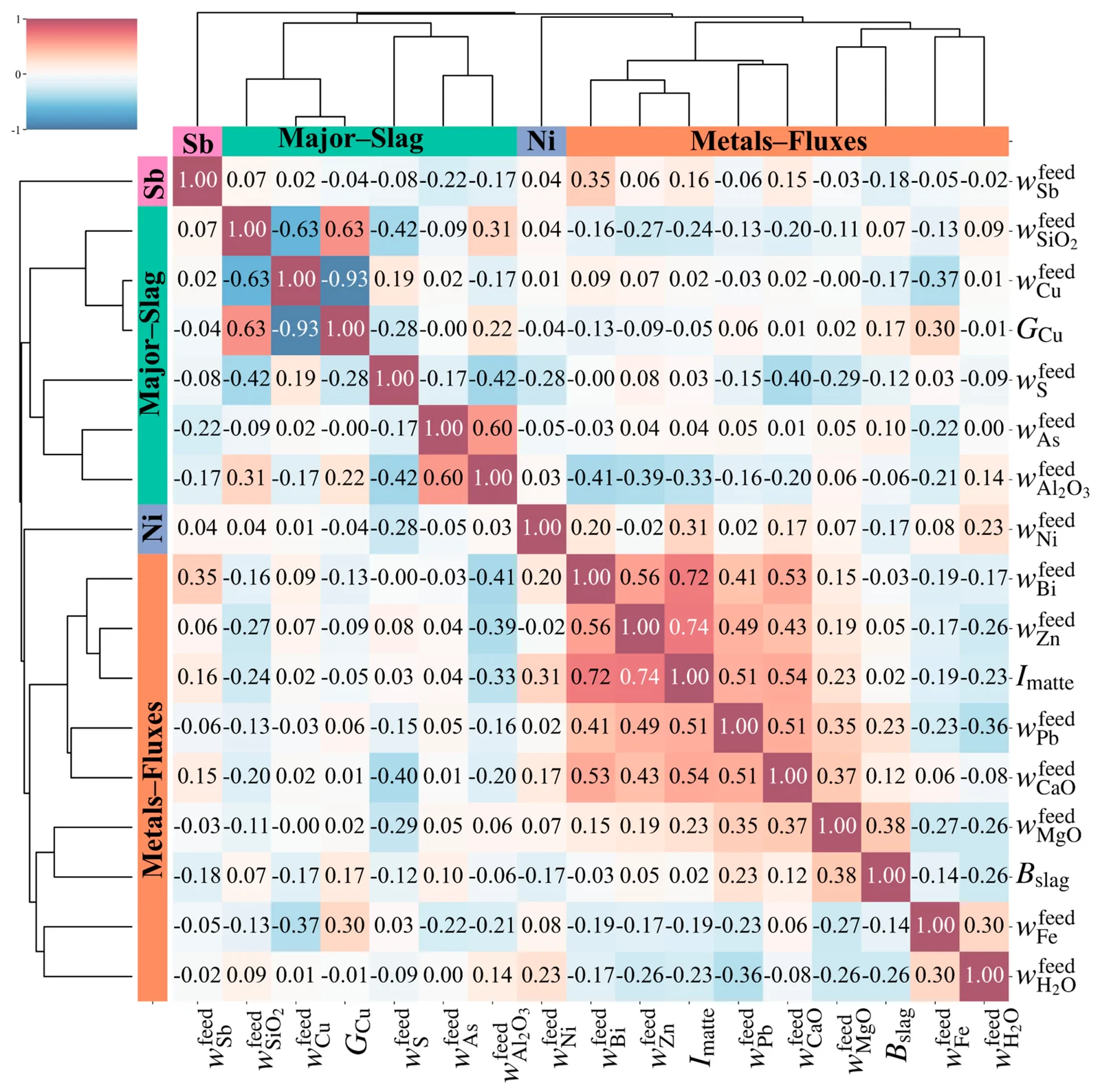
Correlation clustering heatmap of input features and target variables.

After data preprocessing, the input features were finally fixed as $w_{\mathrm{Cu}}^{\mathrm{feed}},w_{\mathrm{Fe}}^{\mathrm{feed}},w_{S}^{\mathrm{feed}},w_{{\mathrm{SiO}}_{2}}^{\mathrm{feed}},w_{\mathrm{As}}^{\mathrm{feed}},w_{\mathrm{Pb}}^{\mathrm{feed}},w_{\mathrm{Zn}}^{\mathrm{feed}},w_{\mathrm{Sb}}^{\mathrm{feed}},w_{\mathrm{Bi}}^{\mathrm{feed}},w_{\mathrm{Ni}}^{\mathrm{feed}},w_{\mathrm{CaO}}^{\mathrm{feed}},w_{{\mathrm{Al}}_{2}O_{3}}^{\mathrm{feed}},w_{\mathrm{MgO}}^{\mathrm{feed}}$, and $w_{H_{2}O}^{\mathrm{feed}}$. The target features were fixed as *G*_Cu_,*I*_matte_, and *B*_slag_. The Pearson correlation coefficient matrix was calculated for the input features together with the three target features on the training set. Figure 5 shows the feature correlation clustering heatmap. It reveals the main coupling structure of the simultaneous changes in copper enrichment and associated metals. Through hierarchical clustering of both rows and columns, the variables are divided into four modules, including Sb class, major-slag class, Ni class, and metals-fluxes class. In metals-fluxes, *I*_matte_ is positively correlated with $w_{\mathrm{Zn}}^{\mathrm{feed}},w_{\mathrm{Bi}}^{\mathrm{feed}},w_{\mathrm{CaO}}^{\mathrm{feed}}$ and $w_{\mathrm{Pb}}^{\mathrm{feed}}$, among which the correlation with $w_{\mathrm{Zn}}^{\mathrm{feed}}$ and $w_{\mathrm{Bi}}^{\mathrm{feed}}$ is the strongest. In major-slag, *G*_Cu_ is strongly negatively correlated with $w_{\mathrm{Cu}}^{\mathrm{feed}}$ and positively correlated with $w_{{\mathrm{SiO}}_{2}}^{\mathrm{feed}}$, while $w_{\mathrm{Cu}}^{\mathrm{feed}}$ is negatively correlated with $w_{{\mathrm{SiO}}_{2}}^{\mathrm{feed}}$. Additionally, $w_{\mathrm{As}}^{\mathrm{feed}}$ is strongly positively correlated with $w_{{\mathrm{Al}}_{2}O_{3}}^{\mathrm{feed}}$, and the other variables are mostly weak to moderate correlations.

## 4. Results and Discussion

### 4.1. Effect of Fusion Model on Predictive and Uncertainty Calibration

For model performance validation, Table 3 compares the prediction results of Partial Least Squares Regression (PLSR), Support Vector Regression with Radial Basis Function kernel (SVR–RBF), Random Forest (RF), eXtreme Gradient Boosting (XGBoost), Multilayer Perceptron (MLP), TMUM, HGBR, and TMUM–HGBR for the three targets. Overall, the conventional baseline models exhibited considerable performance variations across different targets. Among them, MLP showed strong nonlinear fitting capability, whereas HGBR demonstrated more stable predictive performance across the three targets. After fusion, TMUM–HGBR achieved the best performance for the *I*_matte_ and *B*_slag_ while maintaining accuracy comparable to that of the best-performing baseline for the *G*_Cu_, indicating that the fusion strategy effectively exploited the complementary strengths of the two models and improved overall predictive performance and cross-target stability. On this basis, the predictive accuracy and uncertainty calibration performance of TMUM, HGBR, and the TMUM–HGBR fusion model were further analyzed.

For TMUM, Bayesian optimization was performed using the total validation loss as the optimization objective, whereas HGBR was optimized independently for each of the three prediction targets using the three-fold cross-validation RMSE as the objective. Table 4 presents the hyperparameter search spaces and the selected optimal configurations. Based on the optimized parameter settings, the predictive performance of the models was further compared. As shown in Table 5, both HGBR and TMUM–HGBR outperformed TMUM in overall predictive performance, with TMUM–HGBR exhibiting more balanced predictive capability across the three targets. On the validation set, the *R*^2^ values of TMUM–HGBR for *G*_Cu_, *I*_matte_, and *B*_slag_ reached 0.8402, 0.9047, and 0.8438, respectively, all higher than those of HGBR (0.8304, 0.8963 and 0.8306). Because the fusion weights, bias terms and variance-calibration coefficients were estimated on this set, these differences partly reflect the fitting of the fusion parameters; the corresponding test-set comparison is therefore reported with confidence intervals and corrected *p*-values in Table 6.

This advantage was maintained on the test set, where the *R*^2^ for the three targets were 0.8317, 0.8824, and 0.8373, with corresponding RMSE of 0.0785, 0.0601, and 0.0104, respectively. The improvement was most pronounced for *B*_slag_, whose increased from 0.7994 for HGBR to 0.8373 for TMUM–HGBR. For *G*_Cu_, the two models showed comparable performance, and the MAE of HGBR was slightly lower than that of the fusion model. Therefore, the advantage of the fusion strategy was not reflected in every individual metric, but mainly in the improvement and balance of overall multi-target predictive performance. In terms of uncertainty evaluation, TMUM–HGBR achieved lower MPIHW_95_ than HGBR on both the validation and test sets, indicating that the TMUM–HGBR model provided more compact prediction intervals. On the test set, the PICP_95_ for the three targets were 93.6%, 92.7%, and 94.1%, with corresponding MPIHW_95_ of 0.1534, 0.1032, and 0.0218, respectively. Although a certain degree of undercoverage was observed, the overall coverage remained close to the nominal 95% level. TMUM–HGBR provided the most compact intervals among the three models at coverage close to the nominal level, and it improved the point accuracy over both single models; the statistical comparison reported in Table 6 shows, however, that this improvement over HGBR is significant only for *B*_slag_. The fused model is therefore used as a unified surrogate mainly because it provides a single calibrated predictive distribution for the three indicators, rather than because it uniformly improves the point accuracy.

To quantify the contribution of each component, an ablation study was performed on the same independent test set, as shown in Table 7. The two base learners were first evaluated individually and in combination. TMUM alone achieved *R*^2^ values of 0.7864, 0.8313 and 0.7800 for *G*_Cu_, *I*_matte_ and *B*_slag_, HGBR alone achieved 0.8304, 0.8774 and 0.7994, and TMUM–HGBR achieved 0.8317, 0.8824 and 0.8373, respectively. The fused model therefore performed better than the better single model for all three indicators, with the largest gain for *B*_slag_ (from 0.7994 to 0.8373). Replacing the validation-estimated weights with equal weights changed *R*^2^ by no more than 0.007, removing the adaptive gate by no more than 0.0004, and removing the bias correction by no more than 0.006, indicating that these three elements affected the point accuracy only marginally. In contrast, the uncertainty components were decisive: removing the covariance term reduced the 95% coverage from 93.6%, 92.7% and 94.1% to 90.0%, 91.4% and 90.0%, and removing both the variance calibration and the covariance term reduced it to 90.5%, 91.4% and 90.0%, while the interval half-width decreased by 15–18%, as shown in Table 8. The bias correction and the variance calibration therefore mainly ensure consistency between the validation and test intervals, whereas the covariance term is required to avoid under-coverage.

The statistical significance of these differences was assessed by a paired bootstrap procedure (10,000 resamples) and by the Diebold–Mariano test with Holm correction; because the residuals of consecutive records are autocorrelated, a heteroskedasticity– and autocorrelation–consistent variance estimator was used for the test statistic, as shown in Table 6. The fused model outperformed TMUM in MAE for all three indicators (ΔMAE = −0.0082, −0.0087 and −0.0018; Holm-adjusted *p* = 0.007, 0.001 and <0.001) and in *R*^2^ for *I*_matte_ (Δ*R*^2^ = +0.0511; *p* = 0.030). Compared with HGBR alone, the improvement was statistically significant only for *B*_slag_ (Δ*R*^2^ = +0.0379, 95% CI 0.0141–0.0690; *p* = 0.050), whereas for *G*_Cu_ and *I*_matte_ the two models were statistically indistinguishable (Δ*R*^2^ = +0.0013 and +0.0050; *p* = 0.85 and 0.22). The benefit of the hybrid model is therefore target-dependent: it is substantial for the slag basicity indicator, for which the two learners provide complementary information, and marginal for the copper grade enrichment ratio and the composite impurity index, for which HGBR carries most of the fusion weight (0.80 and 0.77, respectively). For the latter two indicators, the main contribution of the fusion is the calibrated predictive distribution rather than the point estimate. Table 9 further reports the random 5-fold cross-validation results, in which the mean *R*^2^ values were 0.878, 0.926, and 0.792 for *G*_Cu_, *I*_matte_, and *B*_slag_, with standard deviations of no more than 0.034 across folds; the model was therefore stable with respect to sample resampling.

**Figure 6:**
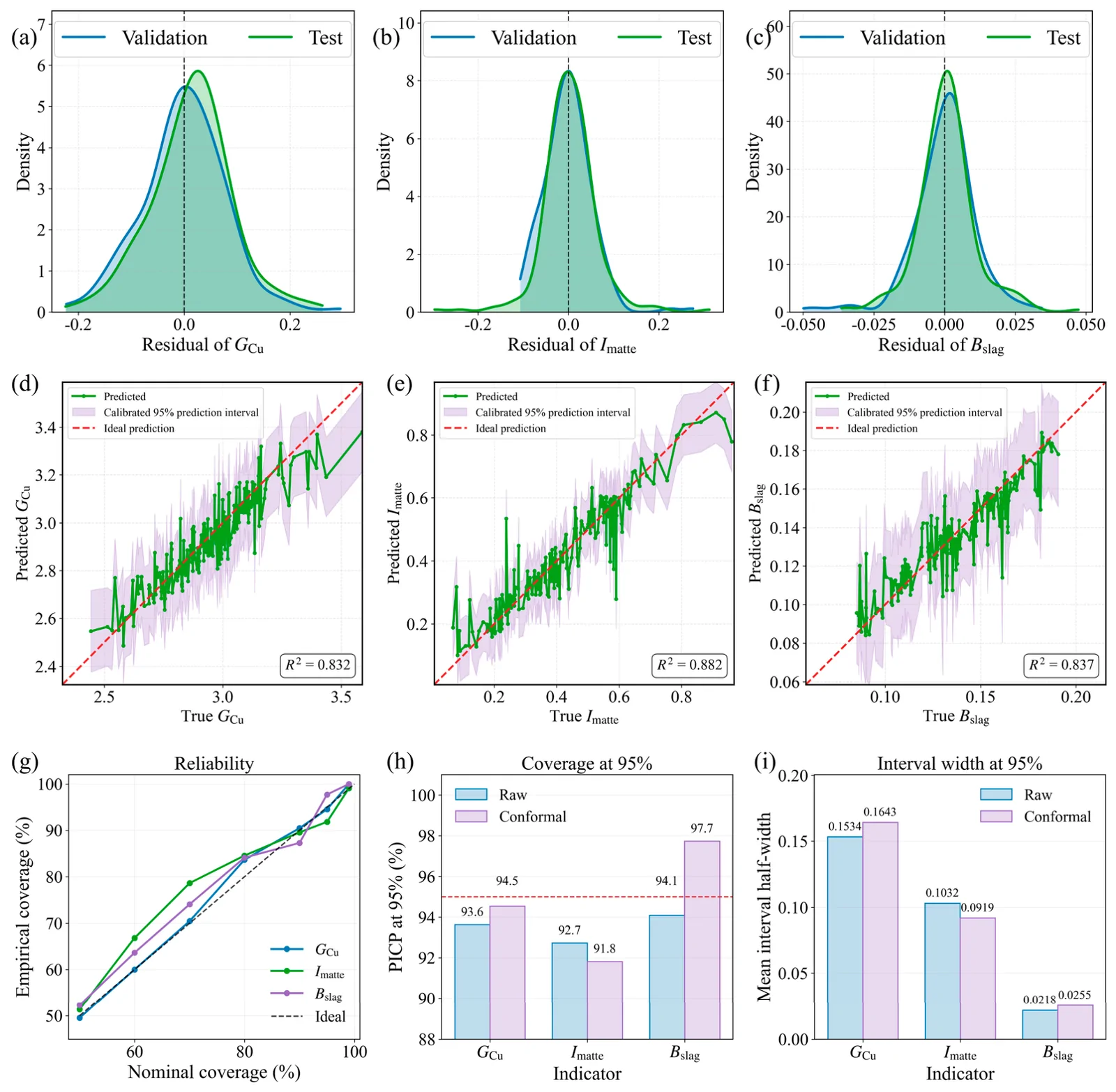
Residual distributions, prediction intervals and calibration of the TMUM–HGBR model. (**a**) Residual distributions of *G_Cu_*; (**b**) Residual distributions of *I*_matte_; (**c**) Residual distributions of *B*_slag_; (**d**) Prediction intervals of *G_Cu_*; (**e**) Prediction intervals of *I*_matte_; (**f**) Prediction intervals of *B*_slag_; (**g**) reliability curves of the three targets at nominal coverage levels from 50% to 99%; (**h**) empirical coverage of the raw and conformal intervals at the 95% nominal level; (**i**) mean prediction–interval half–width of the raw and conformal intervals at the 95% nominal level.

Figure 6 further analyzes the prediction behavior of TMUM–HGBR from the perspectives of residual distributions and sample–level prediction results. Figure 6a–c show the residual density distributions of *G*_Cu_, *I*_matte_, and *B*_slag_ on the validation and test sets, respectively. The residuals of all three targets were mainly concentrated around zero, and the validation and test distributions showed a high degree of overlap, indicating that the model exhibited similar error structures on both datasets without obvious distributional shifts. Among the three targets, the residual distribution of *I*_matte_ had a peak closest to zero and was relatively symmetric. The residuals of *B*_slag_ were highly concentrated on its own scale, although a small number of tail errors remained on both sides. For *G*_Cu_, the peak of the test–set residual distribution showed a slight deviation from zero, suggesting that a certain degree of systematic bias remained for some samples. Figure 6d–f further present the observed values, predicted values, and calibrated 95% prediction intervals for the test samples. The predictions of the three targets generally followed the ideal prediction line, indicating that the model effectively captured the overall variation trends of the target variables across samples, with *I*_matte_ showing the highest agreement between predicted and observed values. At both ends of the value ranges, the predictions exhibited a certain degree of shrinkage toward the central region, where relatively low observed values tended to be slightly overestimated, whereas relatively high observed values tended to be underestimated. This phenomenon was more evident for *G*_Cu_ and *B*_slag_. The prediction intervals were relatively narrow in the central regions with dense samples and widened near the boundaries and in data–sparse regions, indicating that the estimated uncertainty responded, to some extent, to variations in prediction difficulty across different regions of the sample space. Figure 6g–i further evaluate the calibration of the prediction intervals. At nominal levels from 50% to 99%, the empirical coverage of the three targets followed the nominal level closely, with mean absolute calibration errors of 0.92%, 3.58% and 2.94% and root–mean–square calibration errors of 1.47%, 4.69% and 3.11% for *G*_Cu_, *I*_matte_ and *B*_slag_, respectively. With split conformal calibration based on normalized nonconformity scores estimated on the validation set, the test coverages at the 95% level were 94.5%, 91.8% and 97.7% with mean half–widths of 0.1643, 0.0919 and 0.0255, compared with 93.6%, 92.7% and 94.1% and half–widths of 0.1534, 0.1032 and 0.0218 for the raw intervals. Conformal calibration therefore adjusted the interval width but did not systematically eliminate the residual under–coverage of *I*_matte_ and the over–coverage of *B*_slag_, indicating that the remaining deviation is associated with the temporal shift in the test period rather than with a defect of the uncertainty model itself.

**Figure 7:**
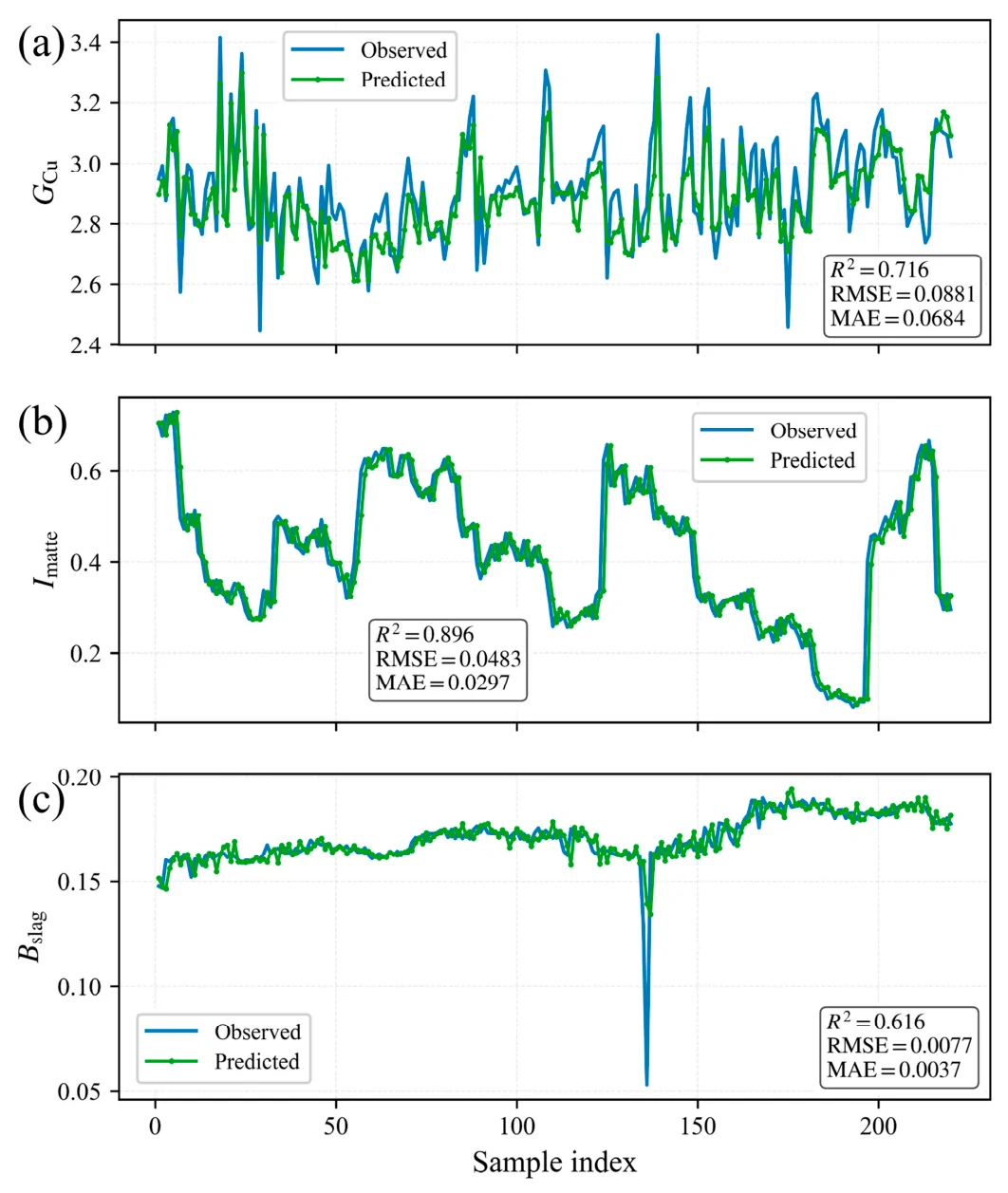
Prediction performance of the TMUM–HGBR fusion model under time–sequential validation. (**a**) *G*_Cu_; (**b**) *I*_matte_; (**c**) *B*_slag_.

To further evaluate the generalization ability of the model under temporal extrapolation, all 1098 samples were arranged in strictly ascending chronological order and divided into training, validation, and test sets at approximately 64%, 16%, and 20%, containing 702, 176, and 220 samples, respectively. Samples with identical timestamps were assigned to the same subset to avoid future information leakage. The prediction results obtained under this strict chronological split are shown in Figure 7. TMUM–HGBR achieved *R*^2^ of 0.716, 0.896, and 0.616 for *G*_Cu_, *I*_matte_, and *B*_slag_, respectively, with corresponding RMSE of 0.0881, 0.0483, and 0.0077. Although the prediction difficulty varied among the three targets, the predicted trajectories generally followed the main trends of the observed values. Among them, *I*_matte_ showed the most stable fitting performance. However, the *R*^2^ values obtained under this single temporal hold–out were lower than those obtained under random splitting, particularly for *B*_slag_, indicating that the accuracy of the model depends on the position of the evaluation window in time. This evaluation therefore characterizes one future period rather than the average behavior over successive future windows.

**Figure 8:**
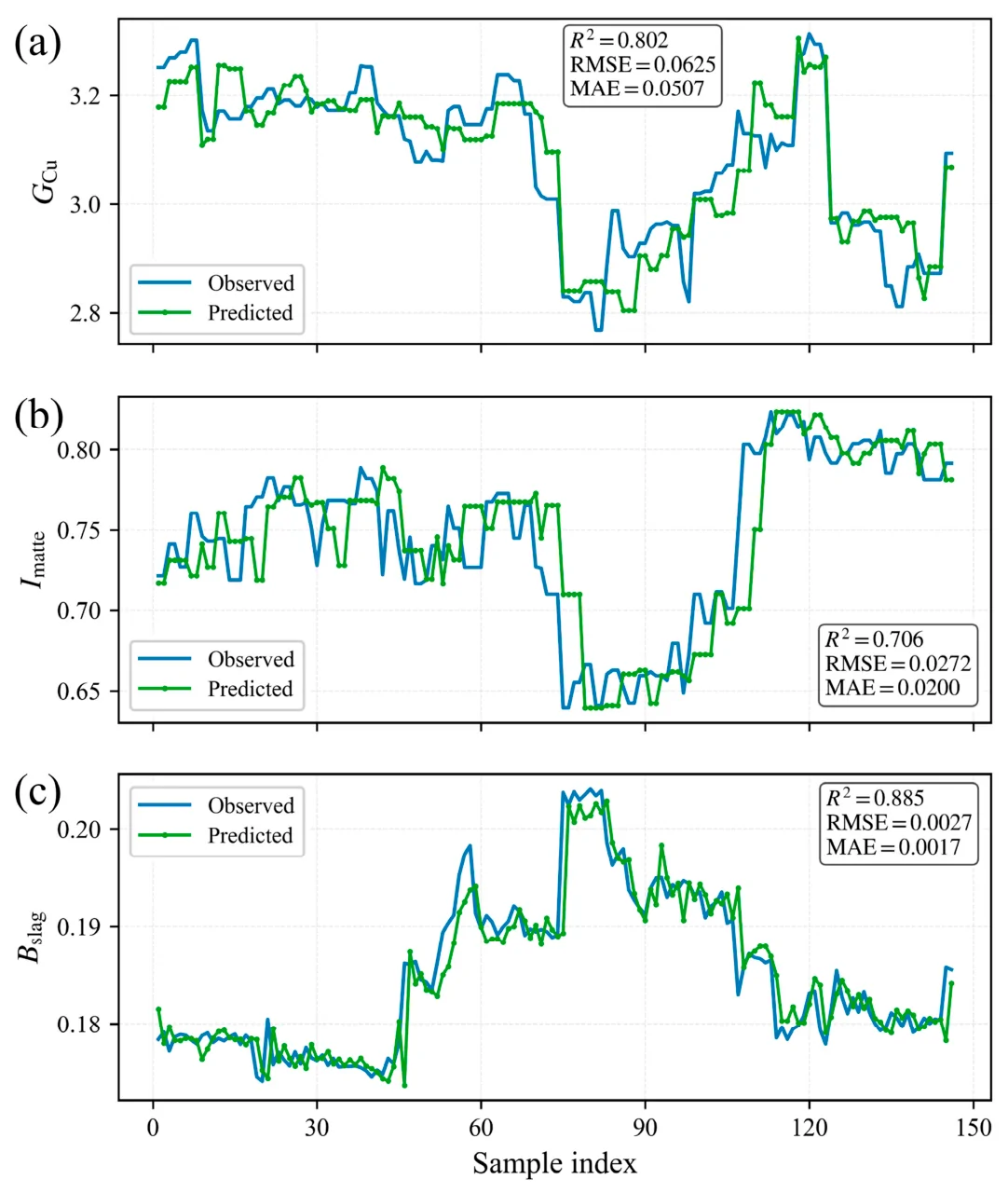
Prediction performance of the TMUM–HGBR fusion model on the external dataset. (**a**) *G*_Cu_; (**b**) *I*_matte_; (**c**) *B*_slag_.

To further examine the model in a prospective setting, newly collected production data that were not involved in training or tuning were used for external validation. The external dataset likewise contained three types of production records: mixed feed, copper matte, and slag. After temporal matching, 469 valid samples were obtained. In chronological order, the first 323 samples were used for calibration and historical–state updating during external validation, while the subsequent 146 samples were used as the final evaluation set. Figure 8 presents the observed and predicted values of the three targets over this evaluation set. Under this operational protocol, the *R*^2^ for the three targets reached 0.802, 0.706, and 0.885, respectively, while the RMSE and MAE of *B*_slag_ were only 0.0027 and 0.0017, respectively. These values characterize a deployment setting with delayed feedback rather than a zero–shot transfer of the frozen model. Taken together, the results from random splitting, random 5–fold cross–validation, chronological validation and the prospective evaluation indicate that the fusion model achieves good accuracy on the data distributions covered in this study, while the accuracy obtained under a single future period is lower, reflecting the effect of temporal drift on extrapolation.

### 4.2. Interpretability and Output Characteristics of the Fusion Model

**Figure 9:**
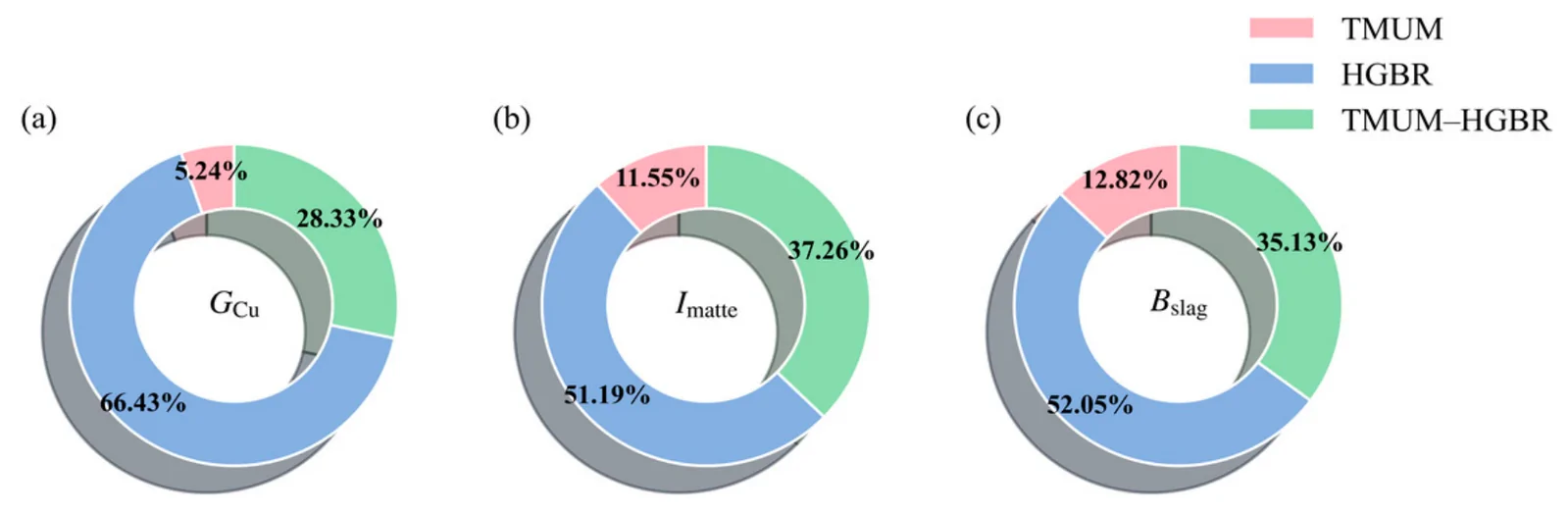
The contribution ratios of the three models on different prediction targets. (**a**) The contribution ratio of *G*_Cu_; (**b**) The contribution ratio of *I*_matte_; (**c**) The contribution ratio of *B*_slag_.

Further elucidating the differences in adaptability of different sub models within the fusion framework to multiple prediction targets, the relative contributions of TMUM, HGBR, and TMUM–HGBR to each target were quantitatively decomposed and analyzed, as shown in Figure 9. For *G*_Cu_, the contribution ratios of HGBR, TMUM–HGBR, and TMUM were 66.43%, 28.33%, and 5.24%, respectively, indicating that the prediction mainly relied on the nonlinear fitting capability of HGBR, while TMUM–HGBR provided additional complementary information. For *I*_matte_, the corresponding contribution ratios of the three models were 51.19%, 37.26%, and 11.55%, respectively. Compared with *G*_Cu_, the contribution of TMUM–HGBR increased markedly, indicating that the prediction of *I*_matte_ relied more strongly on information complementarity among different modeling mechanisms. *B*_slag_ showed a similar pattern, with contribution ratios of 52.05%, 35.13%, and 12.82% for HGBR, TMUM–HGBR, and TMUM, respectively.

**Figure 10:**
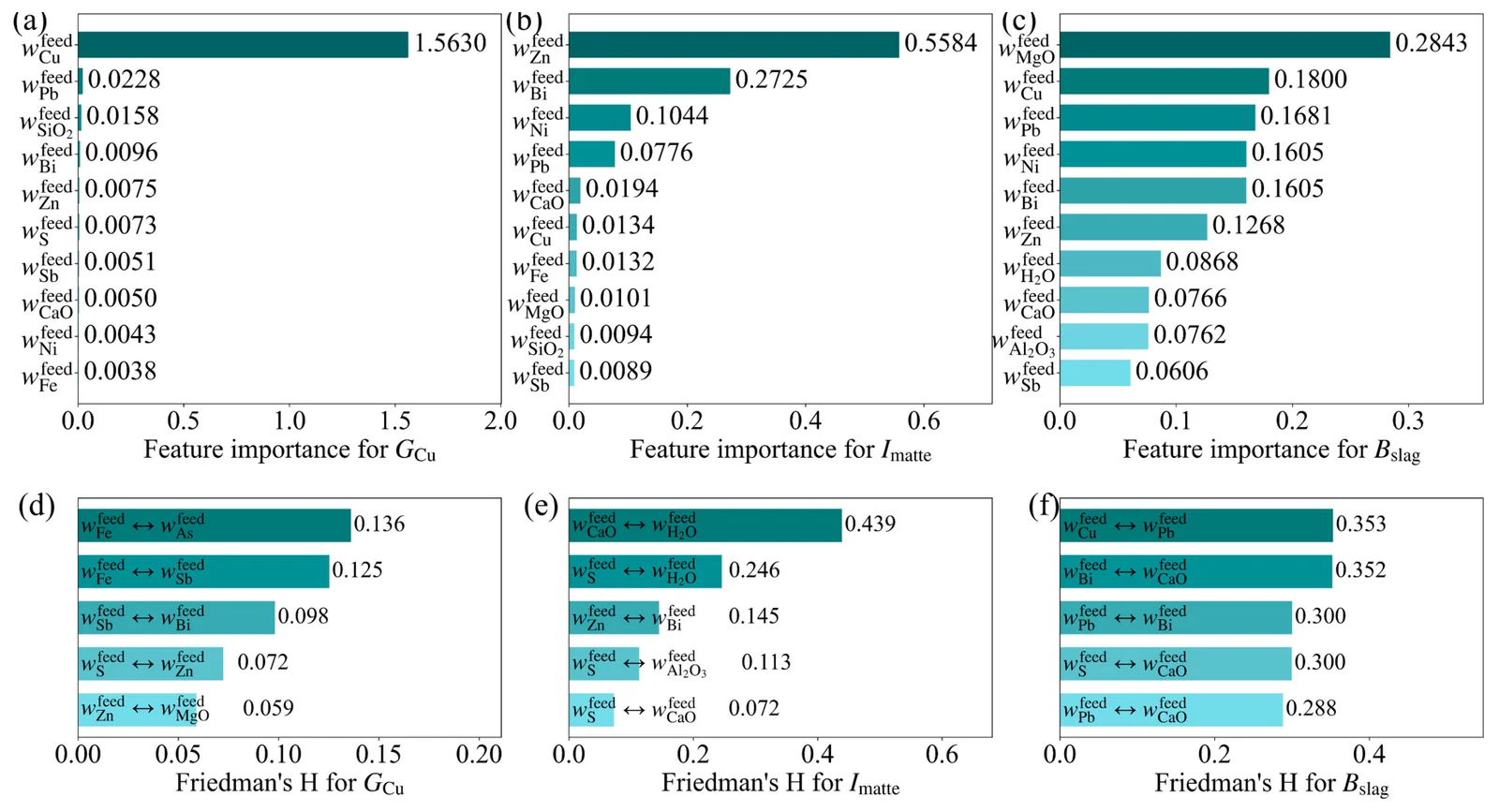
Analysis of the interpretability of the three target variables by the fusion model. (**a**) Ranking of feature importance for *G*_Cu_; (**b**) Ranking of feature importance for *I*_matte_; (**c**) Ranking of feature importance for *B*_slag_; (**d**) Friedman’s H–statistic of pairwise feature interactions for *G*_Cu_; (**e**) Friedman’s H–statistic of pairwise feature interactions for *I*_matte_; (**f**) Friedman’s H–statistic of pairwise feature interactions for *B*_slag_.

To further reveal the basis of the model predictions, the interpretability of TMUM–HGBR was analyzed from the perspectives of global feature contributions and variable interactions, as shown in Figure 10. Figure 10a–c show distinct feature–dependence structures across the three targets. For G_Cu_, $w_{\mathrm{Cu}}^{\mathrm{feed}}$ had the highest importance and substantially exceeded the other variables, indicating that the copper grade of the feed was the primary information source for predicting this target. For *I*_matte_, $w_{\mathrm{Zn}}^{\mathrm{feed}}$ and $w_{\mathrm{Bi}}^{\mathrm{feed}}$ made the largest contributions, with importance values of 0.5584 and 0.2725, respectively, indicating that the impurity index was mainly associated with harmful metal elements in the feed. In contrast, the feature contributions for *B*_slag_ were more dispersed. $w_{\mathrm{MgO}}^{\mathrm{feed}}$ had the highest importance, while $w_{\mathrm{Cu}}^{\mathrm{feed}}$, $w_{\mathrm{Pb}}^{\mathrm{feed}}$, $w_{\mathrm{Ni}}^{\mathrm{feed}}$ and $w_{\mathrm{Bi}}^{\mathrm{feed}}$ also showed relatively high predictive contributions, reflecting a more pronounced multivariable dependence for this target. Figure 10d–f further quantify pairwise feature interactions using Friedman’s H–statistic. For *G*_Cu_, the strongest interactions were Fe–As (H = 0.136), Fe–Sb (0.125) and Sb–Bi (0.098), followed by S–Zn (0.072) and Zn–MgO (0.059), and the mean H over all pairs was only 0.014, consistent with the dominant single–feature structure of this target. For *I*_matte_, the strongest interaction was CaO–H_2_O (H = 0.439), followed by S–H_2_O (0.246), Zn–Bi (0.145), S–Al_2_O_3_ (0.113) and S–CaO (0.073). For *B*_slag_, the strongest interactions were Cu–Pb (0.353), Bi–CaO (0.352), Pb–Bi (0.300), S–CaO (0.300) and Pb–CaO (0.288). Overall, *G*_Cu_ exhibited a strongly single–feature–dominated structure with weak pairwise interactions (mean H = 0.014), whereas *I*_matte_ and *B*_slag_ showed stronger multivariable synergistic characteristics (mean H = 0.025 and 0.054, respectively), indicating differences in the predictive mechanisms of the three metallurgical targets.

**Figure 11:**
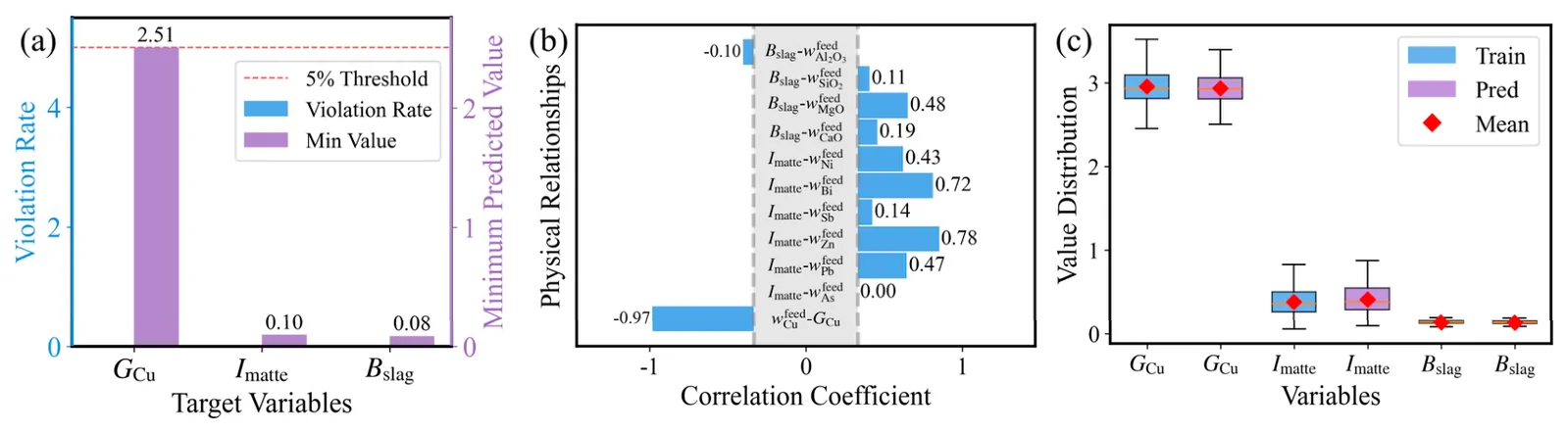
Non–negativity and distributional characteristics of the fusion–model predictions. (**a**) Non–negativity check of the prediction results of the three target variables; (**b**) Correlation analysis between the target variables and key process factors; (**c**) Comparison of the distribution of the training data and the model prediction results.

To further assess the non–negativity, relationship plausibility, and distributional consistency of the model outputs, Figure 11 presents analyses from three perspectives. As shown in Figure 11a, the minimum predicted values of *G*_Cu_, *I*_matte_ and *B*_slag_ were 2.51, 0.10, and 0.08, respectively. No negative predictions were observed for the three targets, indicating that the model outputs satisfied the basic non–negativity requirement. Figure 11b shows target–specific correlations between the different targets and key feed components. *I*_matte_ was positively correlated with most harmful elements in the feed, particularly $w_{\mathrm{Zn}}^{\mathrm{feed}}$ and $w_{\mathrm{Bi}}^{\mathrm{feed}}$, which was consistent with the physical meaning of the impurity index. *B*_slag_ was positively correlated with $w_{\mathrm{MgO}}^{\mathrm{feed}}$ and $w_{\mathrm{CaO}}^{\mathrm{feed}}$, while showing a weak negative correlation with $w_{{\mathrm{Al}}_{2}O_{3}}^{\mathrm{feed}}$. In addition, *G*_Cu_ showed a relatively strong negative correlation with $w_{\mathrm{Cu}}^{\mathrm{feed}}$, which was consistent with its ratio–based definition. Figure 11c shows that the predicted distributions of the three targets were close to those of the training samples in terms of the median, mean, and overall distribution range, indicating that the model largely preserved the main statistical characteristics of the original data during prediction.

**Figure 12:**
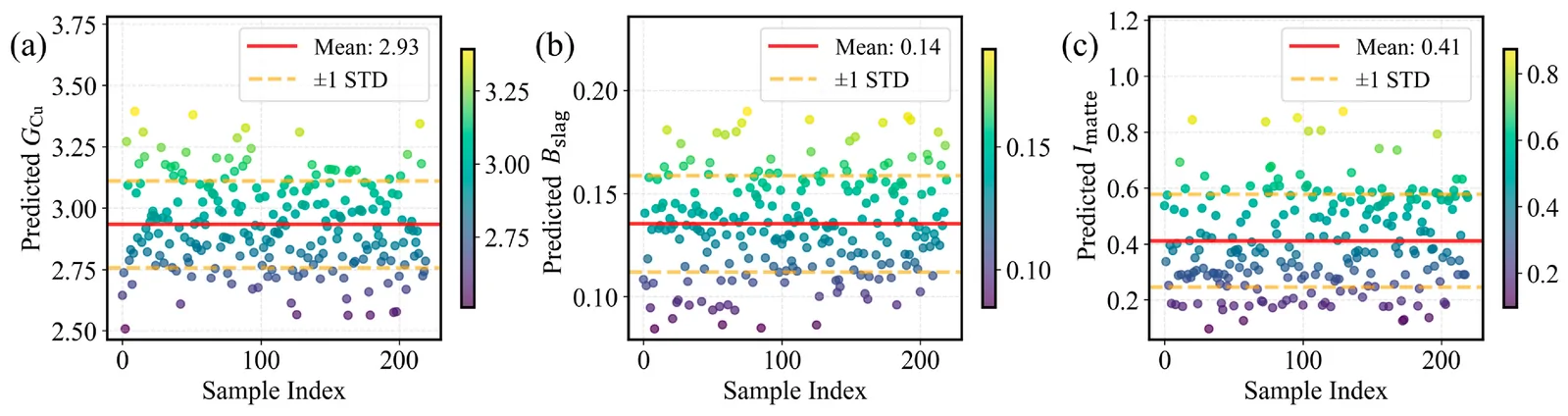
The sample distribution and uncertainty intervals of the prediction results for the three target variables. (**a**) Sample prediction distribution of *G*_Cu_; (**b**) Sample prediction distribution of *B*_slag_; (**c**) Sample prediction distribution of *I*_matte_.

Figure 12 further analyzes the stability and dispersion of the model outputs at the sample level. The mean predicted values of *G*_Cu_, *I*_matte_ and *B*_slag_ were approximately 2.93, 0.41, and 0.14, respectively, with most samples falling within one standard deviation of the mean. *G*_Cu_ exhibited a relatively concentrated prediction distribution with respect to its mean, whereas *I*_matte_ showed greater relative dispersion and more samples in the tail regions, indicating more pronounced sample–to–sample variation in the impurity index. The dispersion of *B*_slag_ was intermediate between those of the other two targets. Overall, the predictions of all three targets remained within plausible ranges without evident abnormal drift, demonstrating good sample–level output stability.

### 4.3. Effect of RVEA on Objective Conflict and Solution Determination

**Figure 13:**
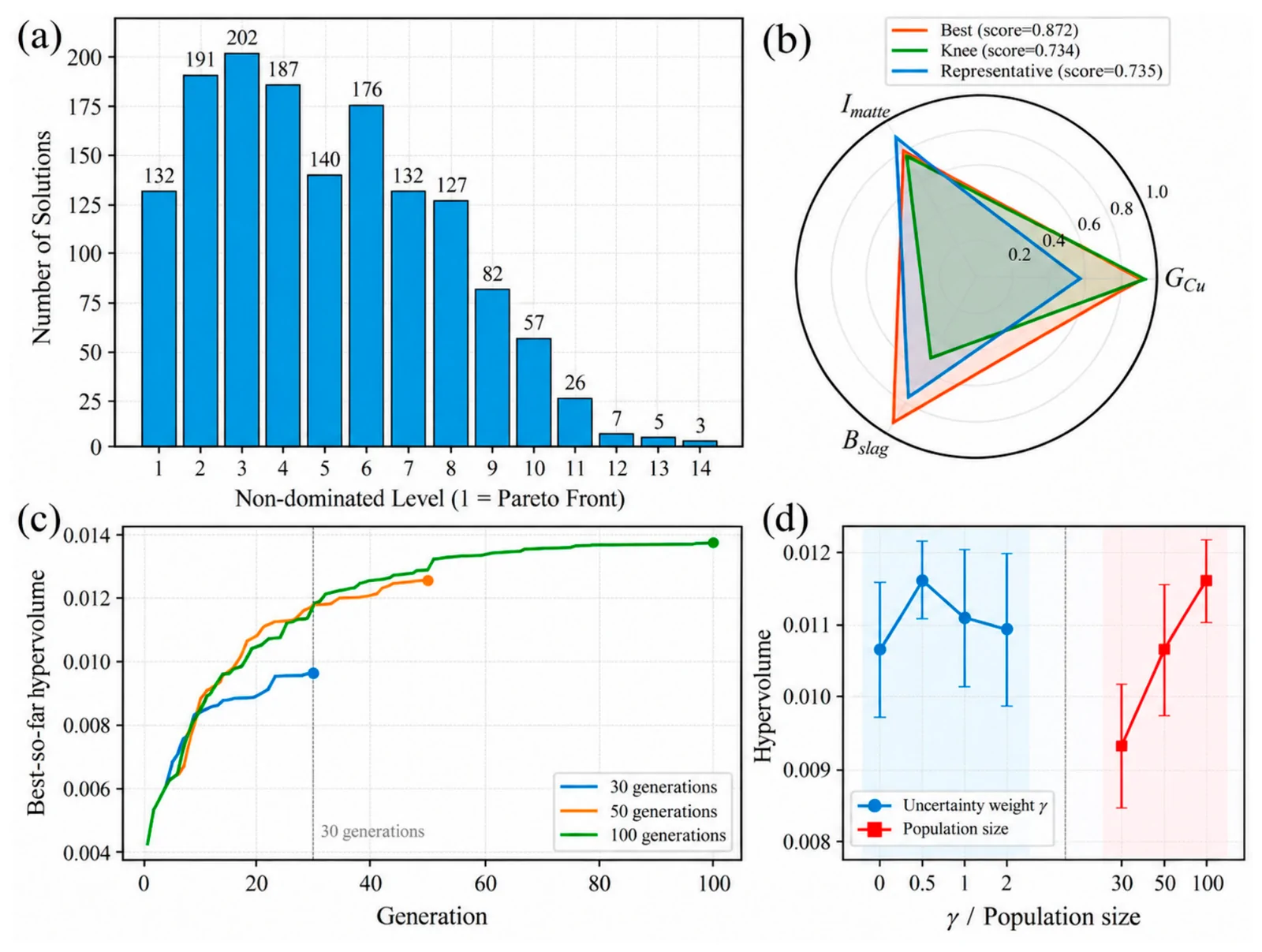
Characteristics of the multi–objective optimization solution set and the convergence/robustness of the algorithm. (**a**) Distribution of solutions across non–dominated levels; (**b**) normalized performance of the optimal, knee–point, and representative solutions; (**c**) best–so–far hypervolume versus generation; (**d**) hypervolume under different uncertainty weights *γ* and population sizes (mean ± standard deviation over three random seeds).

To jointly evaluate copper grade enrichment, matte impurity levels, and deviation from a reference slag basicity, a multi–objective optimization problem was formulated to maximize *G*_Cu_, minimize *I*_matte_, and keep *B*_slag_ as close as possible to the target *B*_target_ = 0.14. Equal weights were assigned to *G*_Cu_ and *I*_matte_ to express an equal preference for copper grade enrichment and impurity reduction. *B*_slag_ was mainly used to maintain process stability around its target value and was assigned a relatively lower priority. Accordingly, the weights of the three objectives were set to *w*_1_ = 0.4, *w*_2_ = 0.4, and *w*_3_ = 0.2. Figure 13a shows the distribution of candidate solutions across different non–dominated levels. All candidate solutions were distributed across 14 non–dominated levels. Level 1 contained 132 solutions, whereas Level 3 contained the largest number, with 202 solutions. Despite some fluctuations among adjacent levels, the number of solutions generally decreased with increasing non–dominated level and declined to only three solution at Level 14. The optimization search therefore generated a diverse set of high–quality solutions while preserving candidate schemes with different objective trade–offs. Figure 13b shows the normalized objective characteristics of three representative solutions. The radar chart further compares the optimal solution, knee–point solution, and representative solution, whose dimensionless comprehensive scores were 0.872, 0.734, and 0.735, respectively. The optimal solution achieved the highest comprehensive score. The score of the representative solution was only 0.137 lower, mainly because its normalized *G*_Cu_ value was substantially lower than that of the optimal solution. The knee–point solution had a comprehensive score 0.138 lower than that of the optimal solution and was nearly identical to that of the representative solution. The three solutions showed relatively small differences along the *I*_matte_ axis, whereas the differences along the *G*_Cu_ axis and the *B*_slag_ axis were considerably larger; specifically, the representative solution was lowest along *G*_Cu_ and the knee–point solution was lowest along *B*_slag_. These differences in the normalized objective values were the main reason for the lower comprehensive scores of the representative and knee–point solutions. These results indicate that the three optimization objectives could not simultaneously reach their individual optima and that different candidate solutions corresponded to different trade–off relationships. Therefore, the optimization results should be evaluated in the form of a Pareto solution set, and the final operating scheme should be selected according to the practical priorities of *G*_Cu_, *I*_matte_ and |*B*_slag_ − 0.14| rather than solely on the extreme value of any single objective. Figure 13c presents the best–so–far hypervolume as a function of generation. The hypervolume increased rapidly during the first 20 generations and then leveled off, reaching 0.0119 after 30 generations and 0.0138 after 100 generations, so that the 30–generation run already captured approximately 86% of the final hypervolume. Figure 13d shows the hypervolume obtained with different uncertainty weights *γ* and population sizes. The hypervolume remained nearly unchanged when *γ* was varied from 0 to 2, whereas a population of 30 gave a lower hypervolume and a population of 100 only a slight improvement over 50. These results indicate that the optimization converged within 30 generations and was robust to the uncertainty weight, while a population of 50 provided a reasonable compromise between solution quality and computational cost.

Table 10 summarizes the corresponding hypervolume values. For *γ* = 0, 0.5, 1, and 2, the hypervolume was 0.0107 ± 0.0009, 0.0116 ± 0.0005, 0.0111 ± 0.0010, and 0.0109 ± 0.0011 (mean ± standard deviation over three random seeds), respectively; the differences were within one standard deviation, confirming that *γ* = 1 is a reasonable setting. For population sizes of 30, 50, and 100, the hypervolume was 0.0093 ± 0.0009, 0.0107 ± 0.0009, and 0.0116 ± 0.0006, respectively. The best–so–far hypervolume at generations 10, 30, 50, and 100 was 0.0084, 0.0119, 0.0129, and 0.0138, respectively. To examine the robustness of the recommended solution, the optimization was repeated with three random seeds. The recommended solution gave *G*_Cu_ = 3.559 ± 0.084, *I*_matte_ = 0.197 ± 0.028, and |*B*_slag_ − 0.14| = 0.006 ± 0.003; the recommended compositions dominated 57–91% of the historical batches in the three–objective space, and their nearest–neighbour distance to the historical operating points (1.03–1.18) remained below the constraint limit of 1.309. Because no plant trial was conducted in this study, the recommended scheme should be implemented with online monitoring and periodic recalibration, and its benefits should be confirmed under industrial operating conditions.

To verify that the optimized solutions were physically consistent, the feasibility of the Pareto solutions was evaluated against the joint constraints. When only the per–variable bounds were applied, 28.8% of the Pareto–optimal solutions fell outside the historical feasible region: the total feed composition ranged from 91.9 to 104.5 wt% and the maximum robust distance (1.388) exceeded the historical 99.5th percentile (1.309). As shown in Table 11, with the joint constraints, all Pareto solutions satisfied the constraint conditions, and the median nearest–neighbour distance to the historical operating points decreased from 1.159 to 1.102. With the additional composition–closure constraint, all solutions satisfied Σw = 100 ± 2 wt% (98.0–101.9 wt%), and the median distance decreased further to 1.059. These results confirm that the optimized compositions remained within the historically observed process region and that the joint constraints were necessary to prevent physically inconsistent extrapolation.

**Figure 14:**
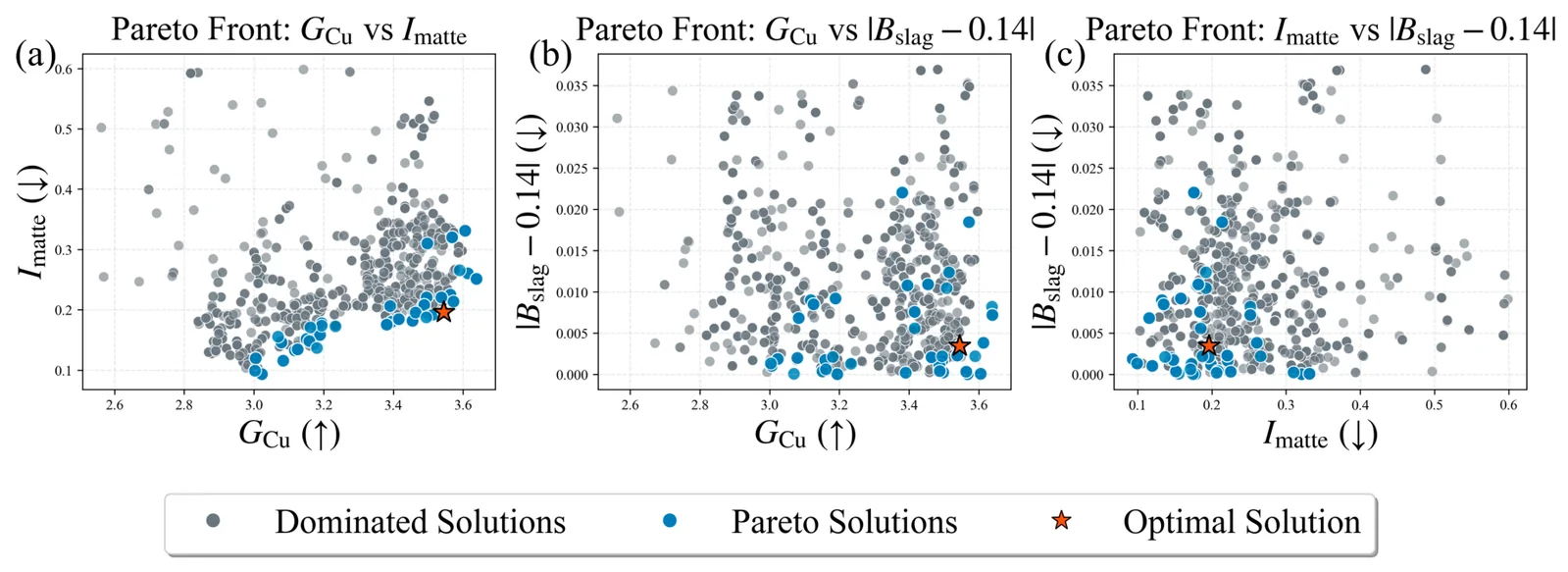
The projection distribution of the Pareto front solution under different target combinations. (**a**) *G*_Cu_–*I*_matte_; (**b**) *G*_Cu_–|*B*_slag_ − 0.14|; (**c**) *I*_matte_–|*B*_slag_ − 0.14|. Gray dots denote dominated solutions, blue dots Pareto solutions, and the star the selected optimal solution; arrows in the axis labels indicate the optimization direction (↑ maximize, ↓ minimize).

Figure 14 further presents the two–dimensional projections of the optimized solutions for each pairwise combination of the three objectives, where the blue points represent Pareto solutions, the gray points represent dominated solutions, and the star denotes the selected optimal solution. As shown in Figure 14a, the Pareto solutions were mainly distributed in regions with relatively high *G*_Cu_ and low *I*_matte_. When *G*_Cu_ was further increased beyond approximately 3.5, the minimum achievable *I*_matte_ increased more markedly, from 0.19 at *G*_Cu_ ≈ 3.5 to 0.25 at *G*_Cu_ ≈ 3.6, indicating a trade–off between further increasing copper grade enrichment and reducing the impurity index. However, several Pareto solutions with low *I*_matte_ were still observed at high *G*_Cu_, suggesting that the two objectives were not completely conflicting. Figure 14b shows the relationship between *G*_Cu_ and |*B*_slag_ − 0.14|. Even in the high–*G*_Cu_ region, Pareto solutions with small or nearly zero basicity deviations were obtained, indicating that increasing *G*_Cu_ did not necessarily cause *B*_slag_ to deviate substantially from the target value of 0.14. Therefore, within the current search space, the conflict between copper grade enrichment and slag basicity stability was relatively weak. Figure 14c shows that most Pareto solutions were concentrated in regions with low *I*_matte_ and small |*B*_slag_ − 0.14|, indicating good compatibility between reducing the impurity index and maintaining slag basicity close to its target value under the identified operating conditions. The selected optimal solution was located near the favorable boundary in all three two–dimensional projections and simultaneously exhibited high *G*_Cu_, low *I*_matte_, and a small *B*_slag_ deviation. Overall, the dominant trade–off among the three objectives was between further increasing *G*_Cu_ and reducing *I*_matte_, whereas control of *B*_slag_ around its target value showed relatively weaker conflicts with the other two objectives.

**Figure 15:**
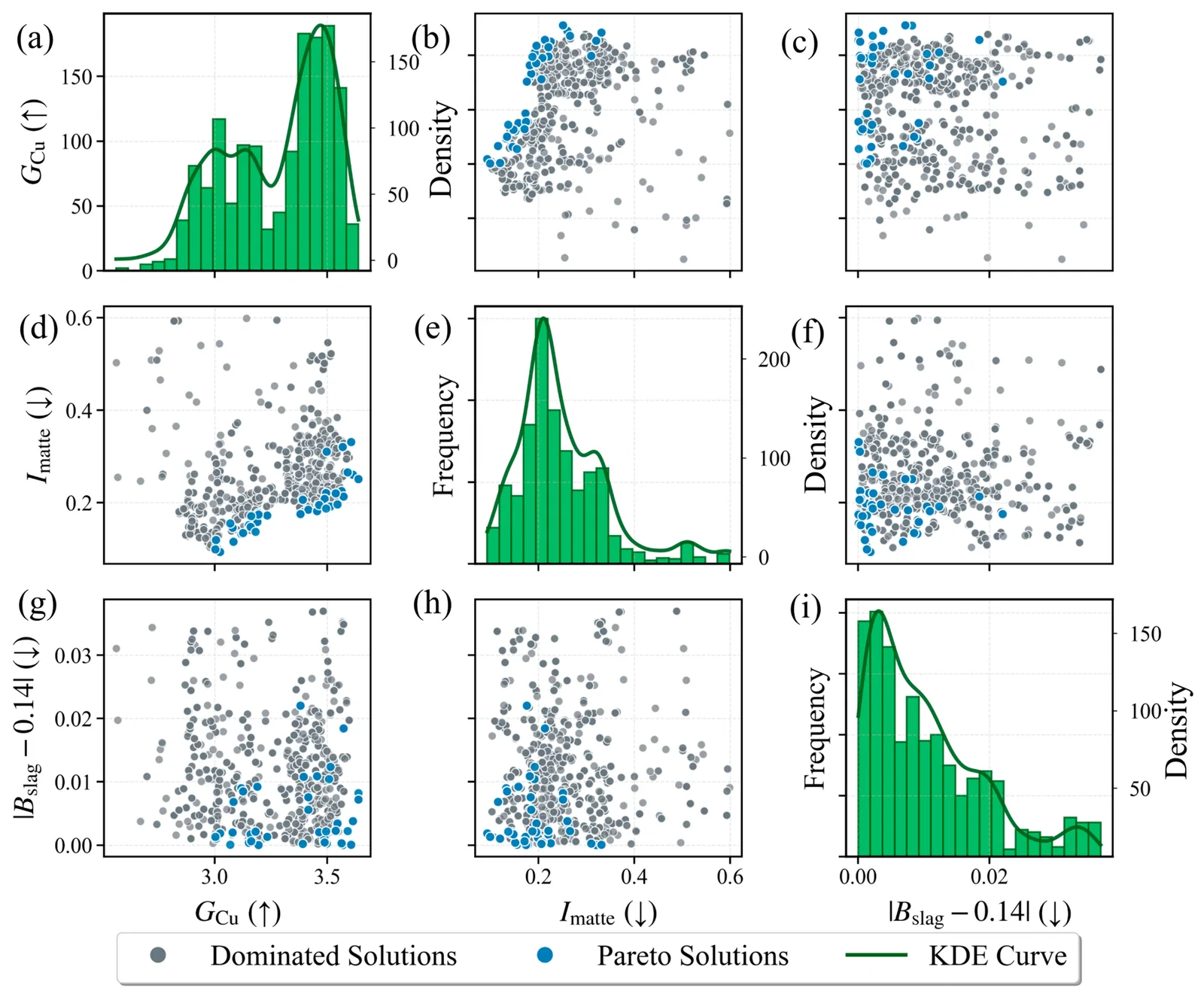
The marginal distribution of the multi–objective optimization solution set and the scatter matrix of pairwise objective relationships. (**a**) Marginal distribution of *G*_Cu_; (**b**) *G*_Cu_ versus *I*_matte_; (**c**) *G*_Cu_ versus |*B*_slag_ − 0.14|; (**d**) *I*_matte_ versus *G*_Cu_; (**e**) marginal distribution of *I*_matte_; (**f**) *I*_matte_ versus |*B*_slag_ − 0.14|; (**g**) |*B*_slag_ − 0.14| versus *G*_Cu_; (**h**) |*B*_slag_ − 0.14| versus *I*_matte_; (**i**) marginal distribution of |*B*_slag_ − 0.14|.

Based on the distribution characteristics of the optimized solution set, Figure 15 further presents the marginal distributions of the three optimization objectives and their pairwise relationships. As shown in Figure 15a, *G*_Cu_ was mainly distributed in the higher–value region, with most solutions above approximately 3.0 and a major density peak around 3.4, indicating that the optimization search retained a considerable number of candidate solutions with relatively high copper enrichment levels. Figure 15e shows that *I*_matte_ was mainly concentrated in the low–value region, although a pronounced right tail remained, suggesting that further reduction in the impurity index was constrained in part of the solution space. In Figure 15i, |*B*_slag_ − 0.14| was highly concentrated near zero, with most deviations below approximately 0.02, indicating that *B*_slag_ remained close to the target value of 0.14 over a relatively broad feasible region. The pairwise scatter distributions further show clear differences between Pareto and dominated solutions in the objective space. Pareto solutions were mainly concentrated in regions with higher *G*_Cu_, lower *I*_matte_, and smaller |*B*_slag_ − 0.14|. In the *I*_matte_ and |*B*_slag_ − 0.14| projection, a large number of Pareto solutions simultaneously maintained low impurity index and small basicity deviations. In contrast, the Pareto solutions in the *G*_Cu_–*I*_matte_ projection were more dispersed along the boundary, reflecting a more pronounced trade–off between increasing copper enrichment and further reducing the impurity index.

**Figure 16:**
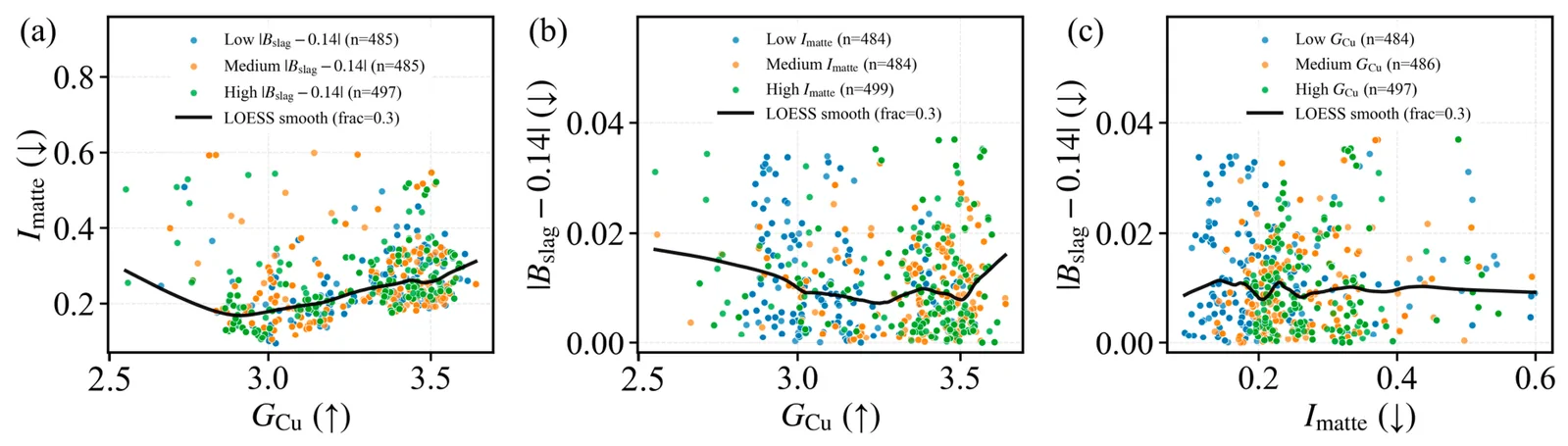
Analysis of the coupling relationships among the three objectives under different target grouping conditions. (**a**) Relationship between *G*_Cu_–*I*_matte_ grouped by |*B*_slag_ − 0.14|; (**b**) Relationship between *G*_Cu_–|*B*_slag_ − 0.14| grouped by *I*_matte_; (**c**) Relationship between *I*_matte_–|*B*_slag_ − 0.14| grouped by *G*_Cu_.

Figure 16 further analyzes the coupling characteristics among the three optimization objectives by grouping the solution set according to the third objective. As shown in Figure 16a, *I*_matte_ first decreased slightly as *G*_Cu_ increased from approximately 2.5 to 2.9, reached a minimum of approximately 0.17 at *G*_Cu_ ≈ 2.9, and then increased steadily when *G*_Cu_ exceeded approximately 2.9, reaching approximately 0.31 at *G*_Cu_ ≈ 3.6. After grouping the solutions according to *B*_slag_−0.14, the low, medium, and high deviation groups still showed substantial overlap in the objective space, indicating that the relationship between *G*_Cu_ and *I*_matte_ was not determined solely by slag basicity deviation. Figure 16b shows a nonlinear relationship between *G*_Cu_ and *B*_slag_−0.14. The basicity deviation decreased from approximately 0.017 at *G*_Cu_ ≈ 2.5 to a minimum of approximately 0.008 at *G*_Cu_ ≈ 3.2, increased again to approximately 0.016 at *G*_Cu_ ≈ 3.6, and remained below 0.02 for most solutions. After grouping by *I*_matte_, the groups largely overlapped over a broad range, and the basicity deviation increased slightly at high *G*_Cu_; nevertheless, increasing *G*_Cu_ did not cause *B*_slag_ to deviate substantially from the target value of 0.14. Figure 16c shows that the basicity deviation remained nearly constant at approximately 0.009 over the range of *I*_matte_, indicating no pronounced dependence of *B*_slag_ on *I*_matte_. After grouping by *G*_Cu_, solutions with high *G*_Cu_ were mainly distributed in regions with higher *I*_matte_ and slightly larger basicity deviations, whereas solutions with low *G*_Cu_ covered broader ranges of both *I*_matte_ and basicity deviation. Overall, the most pronounced trade–off among the three objectives occurred between *G*_Cu_ and *I*_matte_, whereas maintaining *B*_slag_ near 0.14 was relatively compatible with achieving favorable values of the other two objectives.

### 4.4. Interactive Prediction and Feed–Composition Optimization System

**Figure 17:**
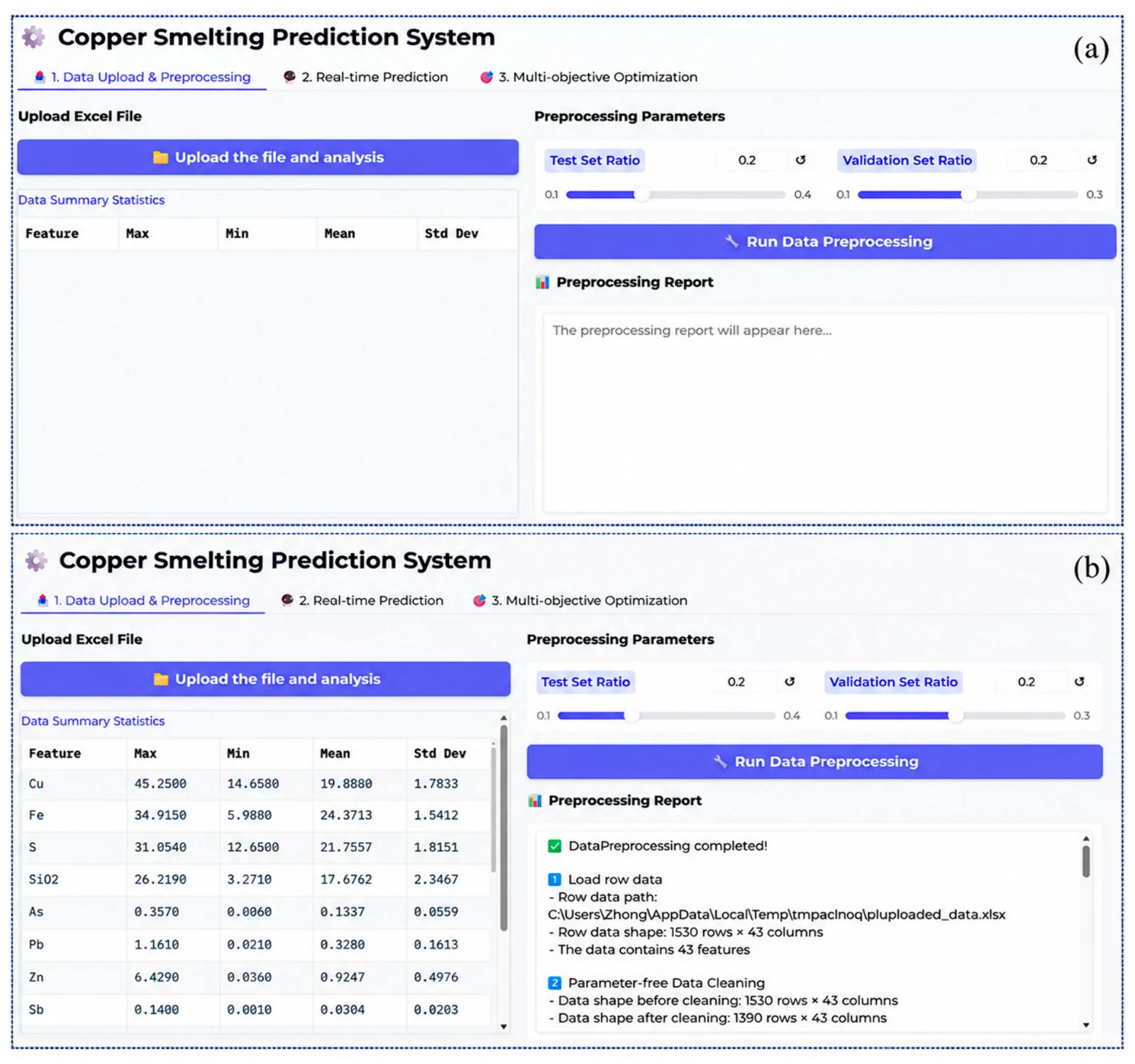
Data upload and preprocessing interface. (**a**) Initial interface; (**b**) Result interface.

An interactive system was developed to integrate indicator prediction and feed–composition optimization for copper smelting. The system was built on the basis of the completed fusion model construction, interpretability analysis, and multi objective optimization study. By integrating the trained fusion model, the TMUM and the HGBR model, the functions of data upload and preprocessing, model prediction, and multi–objective optimization have been achieved through an integrated deployment. Among them, model prediction includes single–sample prediction and batch prediction. With this system, the entire workflow from raw data input to result output and then to process optimization decision making could be completed without directly calling scripts or manually configuring the model environment. Figure 17a shows the interface for data upload and preprocessing. This interface supports uploading raw data and adjust the test set ratio and validation set ratio through sliders. Before the runs, the interface mainly displays the data upload entry, a statistical information area, and a parameter setting area. After the operation, the system outputs a feature statistics table and a preprocessing report, as shown in Figure 17b. The results interface presents the maximum, minimum, mean, and standard deviation of each input variable, and also reports information such as the original data size, the change in sample count before and after cleaning, and the variable dimension.

**Figure 18:**
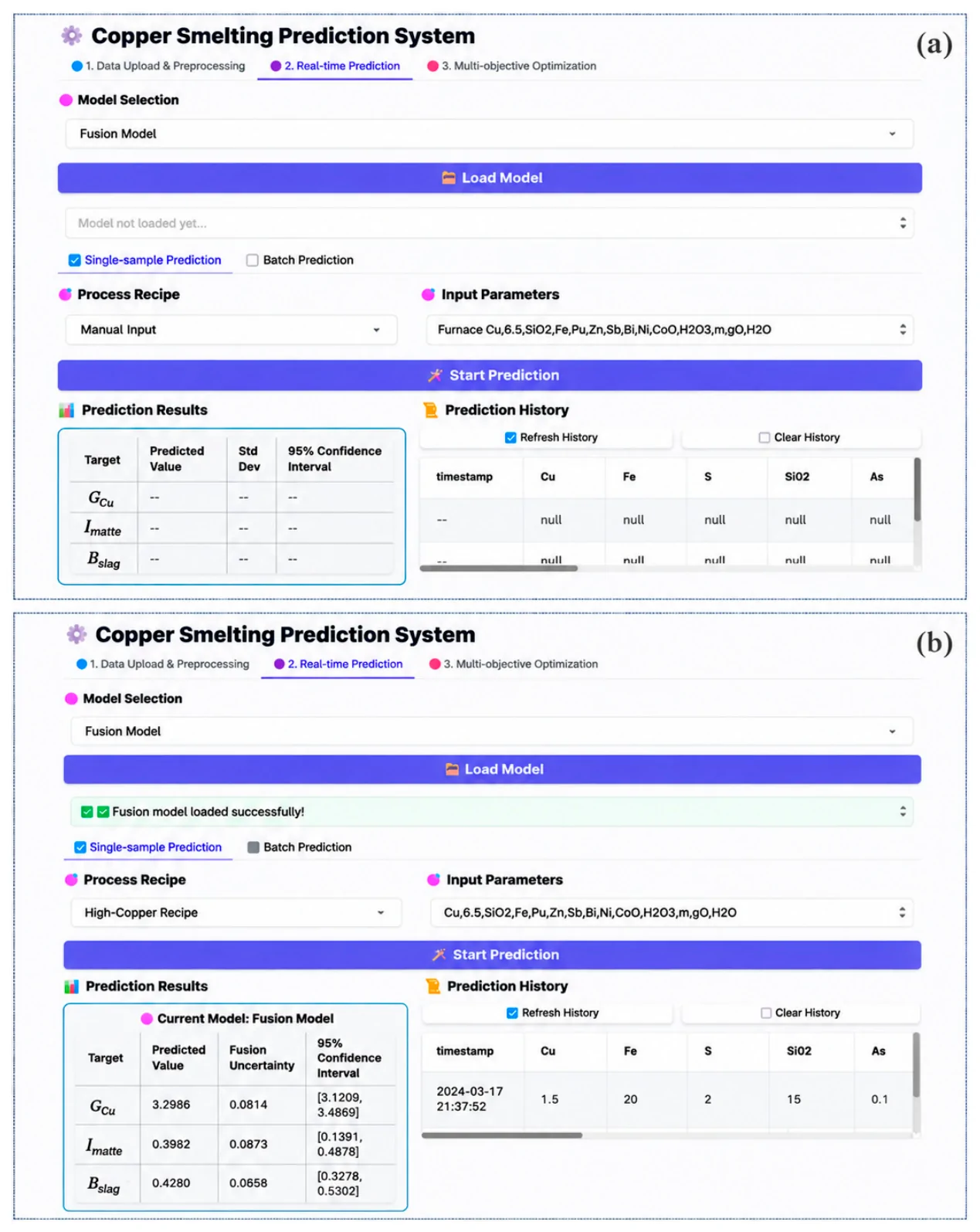
Real–time single–sample prediction interface. (**a**) Initial interface; (**b**) Result interface.

For single sample prediction, an interactive interface for rapid evaluation of process schemes is provided. As shown in Figure 18a, different prediction models can be selected at the top of the interface. In the single sample prediction mode, feed–composition either by manual input or by formula selection. Different methods of the fusion model, TMUM and HGBR models can be selected, enabling the comparison of the predictive performance of different models. After the model is loaded, by inputting or selecting the corresponding formula and starting the prediction. The predicted values of the three target variables, *G*_Cu_, *I*_matte_, and *B*_slag_, are output. As shown in Figure 18b, the results area not only gives the predicted values of the three targets but also outputs the fused uncertainty and the prediction interval half–width simultaneously, and records the current input in the prediction history table.

**Figure 19:**
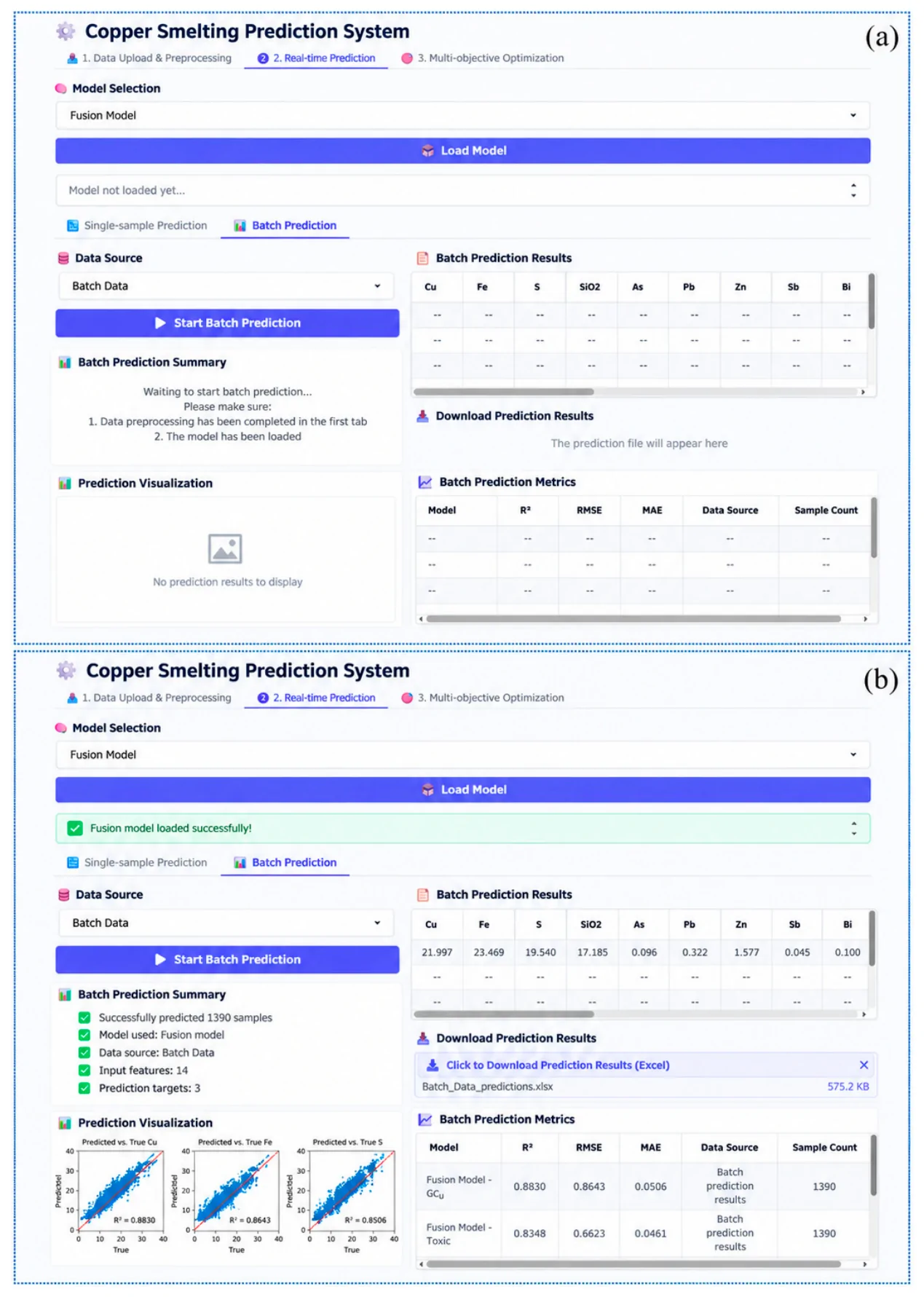
Real–time prediction interface for batch prediction. (**a**) Initial interface; (**b**) Result interface.

In addition to single sample prediction, the batch prediction function for multiple samples of data and the evaluation function using the test set are also supported. As shown in Figure 19a, in the batch prediction mode, the dataset to be predicted can be select and start the calculation for the whole batch of samples. In the initial state of the interface, a batch result table, a result download area, an evaluation metric area, and a visualization area are reserved. After the operation is completed, the batch prediction summary, prediction result table, and download link are output, and automatically generates model evaluation metrics and visualization results, as shown in Figure 19b. The evaluation metrics *R*^2^, RMSE, and MAE are output, and provide a scatter plot comparing the predicted values with the true values to intuitively reflect the fitting effect of the model on each target task. From the results shown in the figure, it can be seen that the predicted scatter points of the three target variables are generally concentrated and distributed near the ideal diagonal, indicating that the encapsulated fusion model still maintains good accuracy and stability in actual batch data analysis.

**Figure 20:**
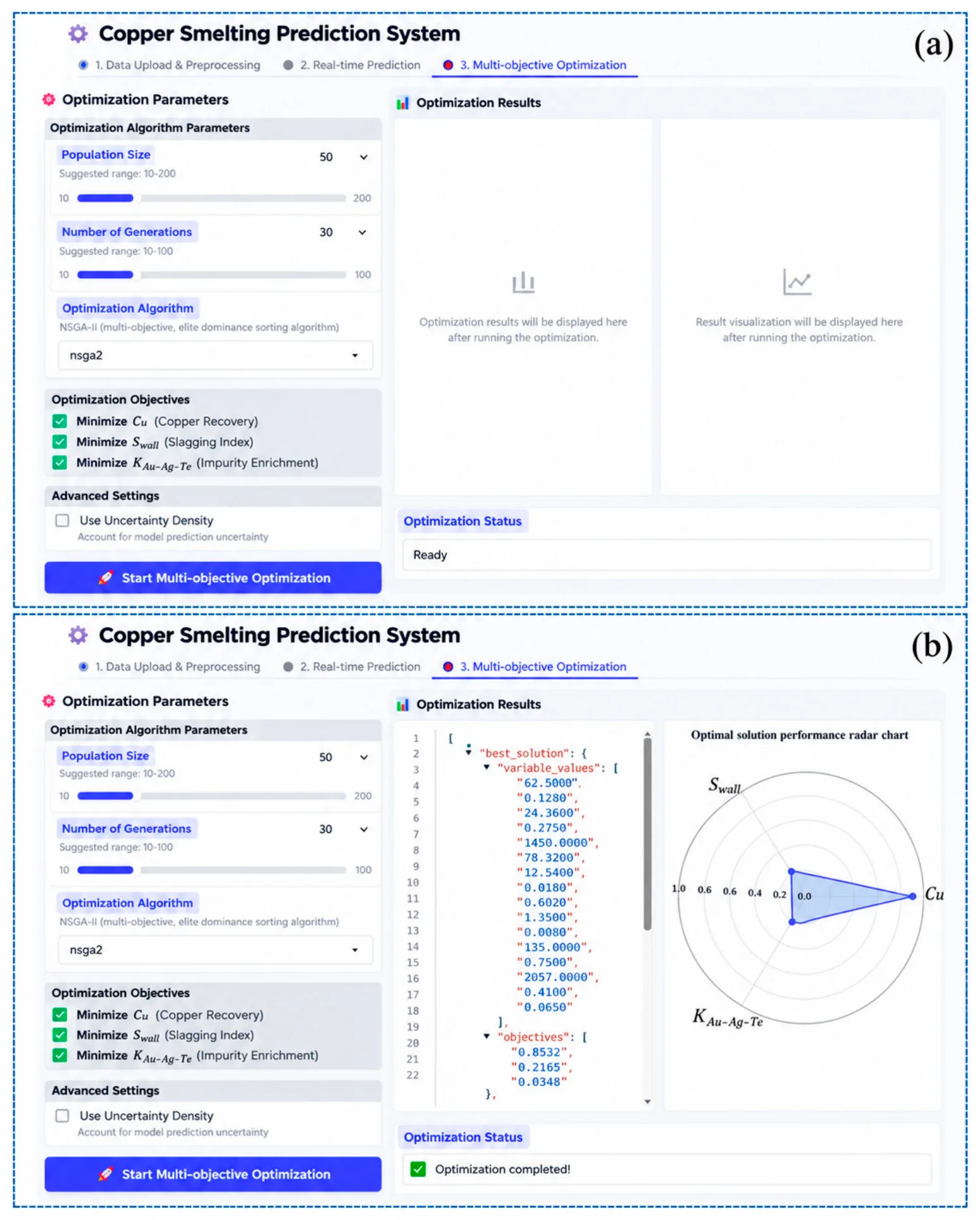
Multi–objective optimization interface. (**a**) Initial interface; (**b**) Result interface.

Further extending the functionality from prediction to decision making, a multi objective optimization module was embedded in the interactive system. As shown in Figure 20a, this module is capable of setting key parameters such as population size, number of generations, and optimization algorithm, and can select different optimization methods as needed. The optimization objectives are consistent with those described earlier. After the parameters are set, the multi objective optimization process can be started. After the optimization ends, the optimal solution results is returned, as shown in Figure 20b, which include the optimal decision variable combination, the corresponding objective function values, and the optimization status information. The comprehensive performance of the three objectives is visualized through a radar chart. The interactive system can not only output static prediction results but also provide interpretable decision support for feed–composition.

The web–based system integrates data preprocessing, indicator prediction, and feed–composition optimization within a single workflow. The system effectively integrates the fusion modeling method, uncertainty quantification strategy, and multi objective optimization framework proposed earlier into a unified platform. On one hand, it preserves the advantages of the models in terms of prediction accuracy, credibility characterization, and optimization decision making. On the other hand, it significantly lowers the usage threshold of the methods through a visual interface. Therefore, this system provides deployable and scalable technical support for key indicator prediction, process scheme evaluation, and parameter optimization in the copper smelting process, and also lays a foundation for the research results to move from theoretical modeling to practical application.

## 5. Conclusions

This work developed an uncertainty–aware framework for multi–indicator soft sensing and multi–objective optimization in copper smelting. The proposed TMUM–HGBR fusion model combines the representation–learning and heteroscedastic uncertainty–estimation capabilities of a Transformer–based multi–task model with the target–specific nonlinear fitting capability of HGBR. Bias correction, variance calibration and covariance–aware uncertainty propagation were further incorporated to construct the predictive distribution. The ablation and the statistical comparison show that the covariance term is the component that materially affects interval coverage, whereas the bias correction and the variance calibration mainly ensure consistency between the validation and test intervals. For the three indicators of copper grade enrichment, composite matte impurity level, and slag basicity, the proposed model achieved test–set *R*^2^ of 0.8317, 0.8824, and 0.8373, respectively, with corresponding 95% prediction–interval coverage probabilities of 93.6%, 92.7%, and 94.1%. Random 5–fold cross–validation confirmed the stability of model under resampling, and a prospective evaluation on newly collected production data indicated that, when the prediction state was updated with available laboratory feedback, the model tracked the three indicators over the evaluation period. The external period showed a level shift in *I*_matte_ and *B*_slag_ relative to the training period, so periodic state updating was required. Interpretability analysis revealed distinct target–dependent feature contributions and interaction patterns while confirming physically plausible prediction behavior. Based on the uncertainty–aware surrogate model, RVEA generated a diverse Pareto solution set for coordinated optimization of the three process objectives. The predicted solutions showed a trade–off between copper enrichment and impurity reduction while keeping slag basicity close to the historical reference of 0.14. Incorporating prediction uncertainty into the optimization objectives reduced preference for high–uncertainty operating regions and improved the reliability of the recommended solutions.

The proposed framework integrates multi–indicator prediction with uncertainty–aware feed–composition optimization. Future work will focus on cross–condition validation, periodic recalibration and adaptive model updating under process drift, incorporation of metallurgical mechanism constraints, and closed–loop online optimization.

## Figures and Tables

**Table 1:** Statistical feature table.

Data	Sample	Features	Unit	Min	Max	Mean	Std
Feed mixture composition	(1149, 14)	$w_{\mathrm{Cu}}^{\mathrm{feed}}$	wt.%	14.6580	45.2580	19.8880	1.7833
		$w_{\mathrm{Fe}}^{\mathrm{feed}}$	wt.%	5.9880	34.9150	24.3713	1.5412
		$w_{S}^{\mathrm{feed}}$	wt.%	12.6500	31.0540	21.7557	1.8151
		$w_{\mathrm{Si}O_{2}}^{\mathrm{feed}}$	wt.%	3.2710	26.2190	17.6762	2.3467
		$w_{\mathrm{As}}^{\mathrm{feed}}$	wt.%	0.0060	0.3570	0.1337	0.0559
		$w_{\mathrm{Pb}}^{\mathrm{feed}}$	wt.%	0.0210	1.1610	0.3280	0.1613
		$w_{\mathrm{Zn}}^{\mathrm{feed}}$	wt.%	0.0360	6.4290	0.9247	0.4976
		$w_{\mathrm{Sb}}^{\mathrm{feed}}$	wt.%	0.0010	0.1400	0.0304	0.0203
		$w_{\mathrm{Bi}}^{\mathrm{feed}}$	wt.%	0.0310	0.1580	0.0719	0.0196
		$w_{\mathrm{Ni}}^{\mathrm{feed}}$	wt.%	0.0060	0.0700	0.0181	0.0093
		$w_{\mathrm{CaO}}^{\mathrm{feed}}$	wt.%	0.3150	4.9880	2.1267	0.4633
		$w_{Al_{2}O_{3}}^{\mathrm{feed}}$	wt.%	0.5240	5.4850	3.1579	0.5193
		$w_{\mathrm{MgO}}^{\mathrm{feed}}$	wt.%	0.0500	2.3960	0.4335	0.2847
		$w_{H_{2}O}^{\mathrm{feed}}$	wt.%	4.1600	13.4400	8.1622	0.9975
Copper matte composition	(3992, 6)	$w_{\mathrm{Cu}}^{\mathrm{matte}}$	wt.%	51.3890	76.2760	58.3108	2.0533
		$w_{\mathrm{Pb}}^{\mathrm{matte}}$	wt.%	0.1480	1.4270	0.5146	0.2252
		$w_{\mathrm{Zn}}^{\mathrm{matte}}$	wt.%	0.0040	3.0220	0.8934	0.6030
		$w_{\mathrm{Sb}}^{\mathrm{matte}}$	wt.%	0.0310	0.1400	0.0920	0.0130
		$w_{\mathrm{Bi}}^{\mathrm{matte}}$	wt.%	0.0150	0.0730	0.0376	0.0107
		$w_{\mathrm{Ni}}^{\mathrm{matte}}$	wt.%	0.0400	0.1670	0.0589	0.0204
Smelting slag composition	(1116, 4)	$w_{\mathrm{Si}O_{2}}^{\mathrm{slag}}$	wt.%	23.0790	33.8160	30.3841	0.8767
		$w_{\mathrm{CaO}}^{\mathrm{slag}}$	wt.%	1.0270	5.1940	3.4517	0.9284
		$w_{Al_{2}O_{3}}^{\mathrm{slag}}$	wt.%	2.5700	5.3340	4.4869	0.3118
		$w_{\mathrm{MgO}}^{\mathrm{slag}}$	wt.%	0.1820	3.2290	1.4194	0.4116

**Table 2:** Entropy-derived weights of the five measured matte impurities.

*i*	1	2	3	4	5
*ω_i_*	0.2018	0.2337	0.2045	0.2182	0.1417
Φ*_i_*	$w_{\mathrm{Zn}}^{\mathrm{matte}}$	$w_{\mathrm{Ni}}^{\mathrm{matte}}$	$w_{\mathrm{Pb}}^{\mathrm{matte}}$	$w_{\mathrm{Bi}}^{\mathrm{matte}}$	$w_{\mathrm{Sb}}^{\mathrm{matte}}$

**Table 3:** Comparison of predictive performance among different models.

Model	*R* ^2^	RMSE	MAE	*R* ^2^	RMSE	MAE	*R* ^2^	RMSE	MAE
Target	*G*_Cu_			*I*_matte_			*B*_slag_		
PLSR	0.7054	0.1039	0.0799	0.6566	0.1027	0.0826	0.2329	0.0226	0.0188
SVR–RBF	0.7558	0.0946	0.0700	0.8067	0.0770	0.0591	0.6118	0.0161	0.0129
RF	0.6384	0.1151	0.0840	0.7088	0.0946	0.0758	0.3610	0.0206	0.0174
XGBoost	0.7671	0.0924	0.0694	0.7367	0.0899	0.0686	0.4009	0.0200	0.0168
MLP	0.8327	0.0783	0.0615	0.8604	0.0655	0.0459	0.7385	0.0132	0.0103
TMUM	0.7864	0.0885	0.0681	0.8313	0.0720	0.0490	0.7800	0.0121	0.0090
HGBR	0.8304	0.0788	0.0595	0.8774	0.0613	0.0413	0.7994	0.0115	0.0077
TMUM–HGBR	0.8317	0.0785	0.0600	0.8824	0.0601	0.0403	0.8373	0.0104	0.0071

**Table 4:** Hyperparameter search spaces and selected optimal configurations for TMUM and HGBR.

Model	Hyperparameter	Type	Search Scope	Optimal Value
TMUM	Encoder dimension *d*_model_	Discrete	{32, 48, 64, 96, 128}	96
	Number of attention heads *h*	Discrete	{2, 4, 8}	4
	Number of encoder layers *L*	Integer range	[2, 5]	4
	Dropout rate	Continuous interval	[0.1, 0.4]	0.2550
	Learning rate	Continuous interval (logarithmic sampling)	[5 × 10^−4^,3 × 10^−3^]	8.64 × 10^−4^
	Weight decay	Continuous interval (logarithmic sampling)	[1 × 10^−6^,3 × 10^−3^]	8.87 × 10^−5^
	Batch size	Discrete	{16, 32, 64}	16
HGBR	Maximum depth of the tree	Integer range	[2, 10]	*G*_Cu_: 2; *I*_matte_: 10; *B*_slag_: 10
	Learning rate	Continuous interval (logarithmic sampling)	[0.01, 0.3]	*G*_Cu_: 0.2386; *I*_matte_: 0.1641; *B*_slag_: 0.0920
	Maximum number of leaf nodes	Integer range	[15, 63]	*G*_Cu_: 62; *I*_matte_: 57; *B*_slag_: 31
	Minimum number of samples at leaf nodes	Integer range	[5, 50]	*G*_Cu_: 6; *I*_matte_: 20; *B*_slag_: 14
	L2 regularization coefficient	Continuous interval (logarithmic sampling)	[1 × 10^−4^,1 × 10^−1^]	*G*_Cu_: 9.03 × 10^−3^; *I*_matte_: 4.75 × 10^−3^; *B*_slag_: 1.31 × 10^−3^

**Table 5:** Performance metrics for models.

Model	Dataset	Target	*R* ^2^	RMSE	MAE	PICP_95_	MPIHW_95_
TMUM	Valid	*G*_Cu_	0.7874	0.0900	0.0683	95.5	0.1849
		*I*_matte_	0.8409	0.0677	0.0488	93.8	0.1203
		*B*_slag_	0.7826	0.0131	0.0092	97.2	0.0250
	Test	*G*_Cu_	0.7864	0.0885	0.0681	95.5	0.1891
		*I*_matte_	0.8313	0.0720	0.0490	93.2	0.1224
		*B*_slag_	0.7800	0.0121	0.0090	95.9	0.0257
HGBR	Valid	*G*_Cu_	0.8304	0.0804	0.0615	93.2	0.1561
		*I*_matte_	0.8963	0.0546	0.0411	97.2	0.1073
		*B*_slag_	0.8306	0.0115	0.0079	95.5	0.0226
	Test	*G*_Cu_	0.8304	0.0788	0.0595	93.2	0.1561
		*I*_matte_	0.8774	0.0613	0.0413	93.6	0.1073
		*B*_slag_	0.7994	0.0115	0.0077	93.2	0.0226
TMUM–HGBR	Valid	*G*_Cu_	0.8402	0.0780	0.0593	93.8	0.1532
		*I*_matte_	0.9047	0.0524	0.0386	98.3	0.1031
		*B*_slag_	0.8438	0.0111	0.0077	95.5	0.0217
	Test	*G*_Cu_	0.8317	0.0785	0.0600	93.6	0.1534
		*I*_matte_	0.8824	0.0601	0.0403	92.7	0.1032
		*B*_slag_	0.8373	0.0104	0.0071	94.1	0.0218

**Table 6:** Statistical comparison between the fused model and the two single models on the independent test set.

Comparison	Target	Δ*R*^2^	95% CI	p(Holm)	ΔMAE	95% CI	p(Holm)
Fusion–TMUM	*G*_Cu_	+0.0453	(0.0115, 0.0848)	0.066	−0.0082	(−0.0133, −0.0033)	0.007
	*I*_matte_	+0.0511	(0.0146, 0.0940)	0.030	−0.0087	(−0.0136, −0.0038)	0.001
	*B*_slag_	+0.0573	(0.0093, 0.1051)	0.063	−0.0018	(−0.0027, −0.0009)	<0.001
Fusion–HGBR	*G*_Cu_	+0.0013	(−0.0095, 0.0127)	0.852	+0.0005	(−0.0017, 0.0027)	0.717
	*I*_matte_	+0.0050	(−0.0010, 0.0114)	0.218	−0.0010	(−0.0026, 0.0006)	0.459
	*B*_slag_	+0.0379	(0.0141, 0.0690)	0.050	−0.0005	(−0.0010, −0.0001)	0.056

**Table 7:** Point–prediction ablation of the TMUM–HGBR model on the independent test set.

Configuration	*G*_Cu_			*I*_matte_			*B*_slag_		
	*R* ^2^	RMSE	MAE	*R* ^2^	RMSE	MAE	*R* ^2^	RMSE	MAE
TMUM	0.7864	0.0885	0.0681	0.8313	0.0720	0.0490	0.7800	0.0121	0.0090
HGBR	0.8304	0.0788	0.0595	0.8774	0.0613	0.0413	0.7994	0.0115	0.0077
Fusion (equal weights)	0.8257	0.0799	0.0609	0.8759	0.0617	0.0421	0.8413	0.0103	0.0073
Fusion (proposed)	0.8317	0.0785	0.0600	0.8824	0.0601	0.0403	0.8373	0.0104	0.0071
Fusion (bias correction)	0.8371	0.0772	0.0582	0.8821	0.0602	0.0407	0.8380	0.0104	0.0071
Fusion (adaptive gate)	0.8317	0.0785	0.0600	0.8820	0.0602	0.0407	0.8373	0.0104	0.0071

**Table 8:** Ablation of the uncertainty components of the TMUM–HGBR model on the independent test set.

Configuration	*G*_Cu_		*I*_matte_		*B*_slag_	
	PICP_95_ (%)	MPIHW_95_	PICP_95_ (%)	MPIHW_95_	PICP_95_ (%)	MPIHW_95_
TMUM	95.5	0.1891	93.2	0.1224	95.9	0.0257
HGBR	93.2	0.1561	93.6	0.1073	93.2	0.0226
Fusion (proposed)	93.6	0.1534	92.7	0.1032	94.1	0.0218
Fusion (without variance calibration)	93.6	0.1542	92.7	0.1029	94.5	0.0220
Fusion (without covariance term)	90.0	0.1299	91.4	0.0858	90.0	0.0176
Fusion (without variance calibration and covariance)	90.5	0.1309	91.4	0.0855	90.0	0.0178

**Table 9:** The 5-fold cross-validation results of the TMUM–HGBR model.

Target	*R*^2^ (Mean ± SD)	RMSE (Mean ± SD)	MAE (Mean ± SD)
*G*_Cu_	0.8778 ± 0.0022	0.0745 ± 0.0022	0.0570 ± 0.0017
*I*_matte_	0.9261 ± 0.0093	0.0493 ± 0.0043	0.0361 ± 0.0030
*B*_slag_	0.7920 ± 0.0342	0.0098 ± 0.0008	0.0070 ± 0.0005

**Table 10:** Convergence and sensitivity of the multi–objective optimization.

Setting	Uncertainty Weight *γ*				Population Size			Generation			
	0	0.5	1	2	30	50	100	10	30	50	100
Hypervolume	0.0107	0.0116	0.0111	0.0109	0.0093	0.0107	0.0116	0.0084	0.0119	0.0129	0.0138
Standard deviation	0.0009	0.0005	0.0010	0.0011	0.0009	0.0009	0.0006	—	—	—	—

**Table 11:** Feasibility of Pareto solutions under different constraint settings.

Setting	Feasible Under Joint Constraints (%)	Σ Within 100 ± 2 wt% (%)	Median Nearest–Neighbour Distance	Hypervolume
Per–variable bounds only	71.2	35.6	1.159	0.01263
Joint constraints	100	48.3	1.102	0.00962
Joint + mass balance (±2 wt%)	100	100	1.059	0.00864

## Data Availability

The data presented in this study are available on request from the corresponding authors with the permission of the factory (third party).

## Abbreviations

The following abbreviations are used in this manuscript:

Abbreviation	Definition
TMUM	Transformer–based Multi–task Uncertainty Model
HGBR	Histogram–based Gradient Boosting Regressor
RVEA	Reference–Vector–Guided Evolutionary Algorithm
RMSE	Root Mean Square Error
MAE	Mean Absolute Error
PICP	Prediction Interval Coverage Probability
MPIW_95_	Mean Prediction Interval Width
TPE	Tree–structured Parzen Estimator
APD	Angle–Penalized Distance
IQR	Interquartile Range
NLL	Negative Log–Likelihood
